# Comparison of (K_0.5_Na_0.5_)NbO_3_ Single Crystals Grown by Seed-Free and Seeded Solid-State Single Crystal Growth

**DOI:** 10.3390/ma16103638

**Published:** 2023-05-10

**Authors:** John G. Fisher, Su-Hyeon Sim, Trung Thành Ðoàn, Eugenie Uwiragiye, Jungwi Mok, Junseong Lee

**Affiliations:** 1 School of Materials Science and Engineering, Chonnam National University, 77 Yongbong-ro, Buk-gu, Gwangju 61186, Republic of Korea; 2 Department of Chemistry, Chonnam National University, 77 Yongbong-ro, Buk-gu, Gwangju 61186, Republic of Korealeespy@chonnam.ac.kr (J.L.)

**Keywords:** (K_0.5_Na_0.5_)NbO_3_, single crystal, solid-state crystal growth, microstructure

## Abstract

(K_0.5_Na_0.5_)NbO_3_-based piezoelectric ceramics are of interest as a lead-free replacement for Pb(Zr,Ti)O_3_. In recent years, single crystals of (K_0.5_Na_0.5_)NbO_3_ with improved properties have been grown by the seed-free solid-state crystal growth method, in which the base composition is doped with a specific amount of donor dopant, inducing a few grains to grow abnormally large and form single crystals. Our laboratory experienced difficulty obtaining repeatable single crystal growth using this method. To try and overcome this problem, single crystals of 0.985(K_0.5_Na_0.5_)NbO_3_-0.015Ba_1.05_Nb_0.77_O_3_ and 0.985(K_0.5_Na_0.5_)NbO_3_-0.015Ba(Cu_0.13_Nb_0.66_)O_3_ were grown both by seed-free solid-state crystal growth and by seeded solid-state crystal growth using [001] and [110]-oriented KTaO_3_ seed crystals. X-ray diffraction was carried out on the bulk samples to confirm that single-crystal growth had taken place. Scanning electron microscopy was used to study sample microstructure. Chemical analysis was carried out using electron-probe microanalysis. The single crystal growth behaviour is explained using the mixed control mechanism of grain growth. Single crystals of (K_0.5_Na_0.5_)NbO_3_ could be grown by both seed-free and seeded solid-state crystal growth. Use of Ba(Cu_0.13_Nb_0.66_)O_3_ allowed a significant reduction in porosity in the single crystals. For both compositions, single crystal growth on [001]-oriented KTaO_3_ seed crystals was more extensive than previously reported in the literature. Large (~8 mm) and relatively dense (<8% porosity) single crystals of 0.985(K_0.5_Na_0.5_)NbO_3_-0.015Ba(Cu_0.13_Nb_0.66_)O_3_ can be grown using a [001]-oriented KTaO_3_ seed crystal. However, the problem of repeatable single crystal growth remains.

## 1. Introduction

Ceramics based on (K_0.5_Na_0.5_)NbO_3_ (KNN) have been extensively studied over the last twenty years as replacements for lead-based piezoelectric ceramics such as Pb(Zr,Ti)O_3_ and Pb(Mg_1/3_Nb_2/3_)O_3_ [1,2]. The base KNN material has only moderate piezoelectric properties (d_33_ = 70–90 pC/N, k_p_ = 0.36–0.39, k_t_ = 0.4, k_33_ = 0.51) [3,4]. Considerable improvements in properties have been achieved by shifting and/or merging the temperatures of the rhombohedral-orthorhombic and orthorhombic-tetragonal phase transitions to/at approximately room temperature [2,5,6,7,8]. Properties have also been improved by preparing KNN and KNN-based single crystals [9,10]. The use of single crystals allows the poling direction of the sample to be oriented in the crystallographic direction that has the best properties [11,12,13,14,15]. Domain engineering and ac poling techniques can also be used to improve the properties [16,17,18]. Single crystals of KNN-based materials have been prepared by methods such as flux growth, top-seeded solution growth, Bridgman growth and solid-state crystal growth [9,12,14,15,19,20,21,22].

In the solid-state crystal growth (SSCG) method, a piece of a single crystal (the seed crystal) is enclosed in a pellet of ceramic powder. The pellet is sintered, and a single crystal of the ceramic grows on the seed crystal [23,24,25]. The SSCG method has several advantages over conventional solution-growth techniques such as lower operating costs (due to the use of standard laboratory furnaces, obviating the need for specialised crystal growth furnaces and expensive Pt crucibles), reduced processing times, lower processing temperatures, and improved composition control [25]. KNN and KNN-based single crystals several millimetres in size have been grown by this method, the size being limited by the size and crystallographic orientation of the seed crystal [12,22,26,27,28,29,30,31,32,33]. However, using the SSCG method to grow KNN single crystals also has some disadvantages. The only suitable seed crystal found so far for KNN is KTaO_3_, which is expensive [34]. Single-crystal growth in the [001] direction is very slow, necessitating the use of [110]-oriented seed crystals [22,31]. This is inconvenient, as the best piezoelectric properties are found in the [001] direction [12,35,36]. Also, for KNN single crystals, the size of the grown crystal is limited by the size of the seed crystal because single crystal growth essentially stops once the rapidly growing (110) face has grown itself out of existence [22]. This also means that not all of the ceramic sample can be converted into a single crystal. Abnormal grain growth in the matrix can impede the growth of the single crystal and the single crystals are often very porous [26,27,31,37,38].

To overcome the limitation of having to use KTaO_3_ seed crystals, work has been carried out by several groups on seed-free solid-state crystal growth (seed-free SSCG). In this method, KNN-based single crystals are grown without using seed crystals by carefully controlling dopant addition and sintering schedule [39,40,41,42,43]. Grain growth is suppressed in the sample except for a small number of grains which grow rapidly to form abnormal grains. These abnormal grains grow and consume all the matrix grains in the sample, forming single crystals. Single crystals of centimetre size have been grown with good piezoelectric properties in KNN and KNN-based systems [39,40,41,42,43,44,45,46,47,48,49,50,51]. KNN-based single crystals grown by this method were successfully used to prepare an intravascular photoacoustic probe [52] and show potential for use as actuators [46,53].

Although seed-free SSCG has the advantage of not needing seed crystals, it also has some limitations. It is difficult to control the number and size of single crystals that grow in each sample, as the formation of abnormal grains takes place randomly. Morimoto et al. were able to limit the single-crystal growth of KNN to one crystal per sample by careful control of the alkali/niobium ratio and the amount of CuO and Bi_2_O_3_ dopants [43,54]. Sometimes, the grown single crystals are very porous [55]. In addition, work in our laboratory has found problems with the repeatability of the experiments. Single crystals can be grown successfully two or three times from a particular batch of powder, but after that single crystals no longer grow. For seed-free SSCG to take place, the sample has to contain at least one grain which is large enough to grow abnormally into a single crystal [24]. A particular sample may or may not contain such a grain depending upon its grain-size distribution. To overcome this problem, an artificially large grain may be added in the form of a seed crystal i.e., seeded SSCG, more commonly called SSCG. Ha et al. used a KTaO_3_ seed crystal to grow a single crystal of KNN in a composition originally developed for seed-free SSCG [56], but as far as we know no more work has been carried out on this topic. In the present work, we grow single crystals of two previously developed seed-free KNN compositions by both seed-free SSCG and seeded SSCG and compare the single crystals grown by each method.

## 2. Materials and Methods

Powders of composition 98.5 mol% (K_0.5_Na_0.5_)NbO_3_-1.5 mol% Ba_1.05_Nb_0.77_O_3_ (KNBaN) and 98.5 mol% (K_0.5_Na_0.5_)NbO_3_-1.5 mol% Ba(Cu_0.13_Nb_0.66_)O_3_ (KNBaCuN)were prepared by the mixed oxide method. These two compositions were found by Rahman et al. to be suitable for growing single crystals by seed-free SSCG [41]. Raw materials of Na_2_CO_3_ (Acros Organics, Geel, Belgium, 99.5% or Kanto, Tokyo, Japan, 99.8%), K_2_CO_3_ (Alfa Aesar, Heysham, Lancashire, United Kingdom, 99% or Daejung, Siheung-si, Republic of Korea, 99.5%), BaCO_3_ (Alfa Aesar, 99.8%), CuO (Alfa Aesar, 99.7%), and Nb_2_O_5_ (Daejung, 99.9%) were dried in an oven at 250 °C for 5 h to remove any adsorbed moisture. Stoichiometric amounts of the raw materials were ball-milled for 18 h in high-purity (99.9%) ethanol in a polypropylene jar with zirconia balls. Most of the ethanol in the slurries was evaporated by using a hot plate and magnetic stirrer and then the remaining ethanol was removed by drying the pastes in an oven at 80 °C for 24 h. The dried powders were crushed in an agate mortar and pestle and sieved through a 180 µm mesh to remove any agglomerates. The ground powders were calcined in high-purity alumina crucibles with lids at 900 °C for 3 h with heating and cooling rates of 5 °C·min^−1^. The calcined powders were ball-milled for 18 h as before. Separate jars, zirconia balls, mortar and pestles, sieves, and crucibles were used for each powder to avoid cross-contamination with CuO between powders. X-ray diffraction (XRD, Malvern Panalytical Empyrean, Malvern, UK) of the calcined powders was carried out in Bragg–Brentano geometry using CuKα radiation with a scan range of 20–90° 2θ, a step size of 0.026° and a scan speed of 3°·min^−1^. Background removal and pattern smoothing were carried out using Match! (Crystal Impact, Bonn, Germany). Kα_2_ peaks were not removed. The powders were stored in a desiccator.

For seed-free SSCG experiments, 0.5 g of powder was hand-pressed into a pellet in a stainless-steel die of 10 mm diameter. The pellet was then cold-isostatically pressed at 50 MPa. Samples were buried in packing powder in an alumina crucible with a lid and sintered at 1125 °C (KNBaCuN samples) or 1135 °C (KNBaN samples) for between 20–21 h, with heating and cooling rates of 5 °C·min^−1^. For seeded SSCG experiments, a KTaO_3_ seed crystal (MTI Corp, Richmond, CA, USA) with [001] or [110] orientation and dimensions of 2.5 mm × 2.5 mm × 0.5 mm was buried in the centre of 0.6 g powder and hand-pressed into a pellet in a stainless-steel die of 10 mm diameter. The pellet was then cold-isostatically pressed at 50 MPa. Samples were buried in packing powder in an alumina crucible with a lid and sintered at 1125 °C (KNBaCuN samples) or 1135 °C (KNBaN samples) for between 5–20 h, with heating and cooling rates of 5 °C·min^−1^. Separate crucibles were used for each composition to avoid CuO cross-contamination. The KNBaN samples were buried in packing powder of the same KNBaN composition but due to an experimental error, the seeded SSCG KNBaCuN samples were buried in a 98.5 mol% (K_0.5_Na_0.5_)(Nb_0.99_Sb_0.01_)O_3_-1.5 mol% Ba(Cu_0.13_Nb_0.66_)O_3_ packing powder. This may cause the seeded SSCG KNBaCuN single crystals to contain a trace amount of Sb. As a result, the Sb content of both the seed-free and seeded SSCG KNBaCuN single crystals was checked for during chemical analysis.

X-ray diffraction (XRD, Malvern Panalytical Empyrean, Malvern, UK) of the sintered bulk samples was carried out in Bragg-Brentano geometry using CuKα radiation with a scan range of 20–90° 2θ, a step size of 0.026°, and a scan speed of 3 °·min^−1^. Background removal and pattern smoothing were carried out using Match! (Crystal Impact, Bonn, Germany). Kα_2_ peaks were not removed. Single crystal XRD was carried out on selected samples grown by seeded SSCG with [001] KTaO_3_ seeds. For single-crystal XRD, the samples were vertically sectioned and polished to remove the part of the single crystal that contained the KTaO_3_ seed crystal. The polycrystalline regions of the sample were then removed by grinding with SiC paper, leaving only the KNN single crystal. Reflection data were collected using a Bruker APEX-II CCD-based diffractometer (Bruker AXS GmbH, Karlsruhe, Germany) with graphite-monochromated MoKα radiation (λ = 0.71073 Å). The hemisphere of the reflection data was collected as ω scan frames at 0.5°/frame and an exposure time of 5 s/frame. The cell parameters were determined and refined using the APEX2 program [57]. The data were corrected for Lorentz and polarization effects and an empirical absorption correction was applied using the SADABS program [58]. The compound structures were solved by direct methods and refined by full-matrix least squares using the SHELXTL program package [59] and Olex2 [60] with anisotropic thermal parameters for all nonhydrogen atoms. The chemical formula (K_0.5_Na_0.5_)NbO_3_ was used to refine the data.

For microscopy, samples were vertically sectioned with a diamond-wheel saw and polished to a 1 μm finish. The halves of the polished samples were thermally etched and Pt coated for microstructural analysis by scanning electron microscopy (SEM, Hitachi S-4700, Tokyo, Japan) equipped with an energy-dispersive X-ray spectrometer (EDX, Horiba EMAX Energy EX-200, Kyoto, Japan) using standard-less analysis. Grain-size distributions of some of the samples were measured from the micrographs using ImageJ v.1.46 software. The area of the grains was measured and converted to equivalent 2D spherical radii. At least 250 grains were measured for each sample. Porosity in the samples was estimated from the micrographs using ImageJ v.1.46. Electron probe microanalysis (EPMA, JEOL JXA-8530F PLUS, Tokyo, Japan) was carried out on selected single crystals to determine their chemical composition. Samples were polished to a 1 μm finish using diamond paste but not thermally etched. Wavelength-dispersive spectroscopy analysis was carried out using an accelerating voltage of 15 kV. NaAlSi_2_O_6_, KNbO_3_, BaSO_4_, Cu, and Sb were used as standards.

## 3. Results

### 3.1. 98.5 mol% (K_0.5_Na_0.5_)NbO_3_-1.5 mol% Ba_1.05_Nb_0.77_O_3_ Seed-Free Solid-State Crystal Growth

**Figure 1:**
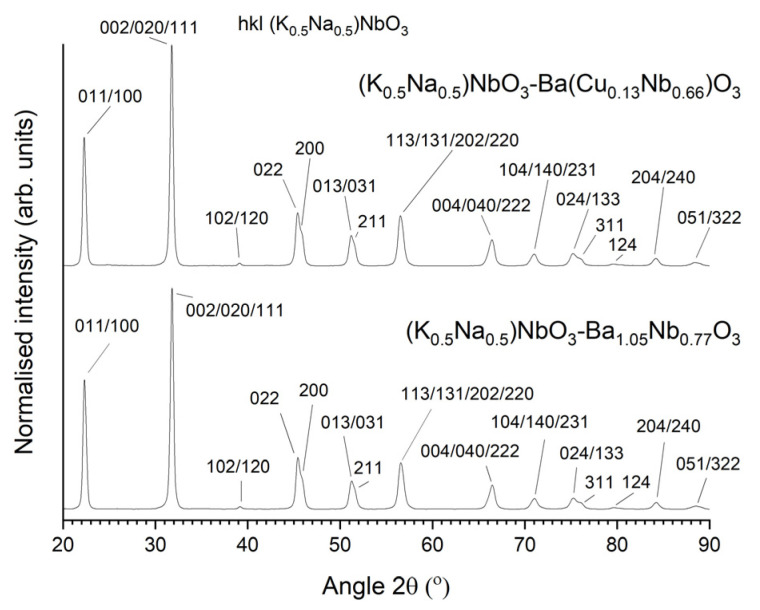
XRD patterns of calcined 98.5 mol% (K_0.5_Na_0.5_)NbO_3_-1.5 mol% Ba_1.05_Nb_0.77_O_3_ and 98.5 mol% (K_0.5_Na_0.5_)NbO_3_-1.5 mol% Ba(Cu_0.13_Nb_0.66_)O_3_ powders.

XRD patterns of the calcined KNBaN and KNBaCuN powders are shown in Figure 1. Both patterns can be indexed with Crystallography Open Database pattern #96-230-0500 for (K_0.5_Na_0.5_)NbO_3_ (orthorhombic, *Amm2*). Both powders appear to be single phase. The peaks are very broad and neighbouring peaks merge together, making identification of individual peaks difficult. The broad peaks indicate a submicron particle size [61]. Both powders had been stored in a desiccator for over 12 months when these XRD patterns were taken, indicating that both powders are stable against the formation of second phases.

**Figure 2:**
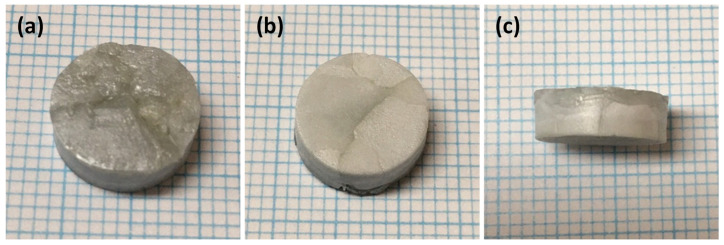
Pictures of a 98.5 mol% (K_0.5_Na_0.5_)NbO_3_-1.5 mol% Ba_1.05_Nb_0.77_O_3_ sample sintered at 1135 °C for 20 h: (**a**) top; (**b**) bottom; (**c**) side. Each blue line = 1 mm.

Single crystals of KNBaN grown by seed-free SSCG at 1135 °C for 20 h are shown in Figure 2. Several single crystals up to ~5 mm in size have grown in the sample. The single crystals that grew from the bottom of the sample are a different colour than those that grew from the top of the sample. The density of a piece of this sample was measured by the Archimedes method in deionised water. The density value (mean and standard deviation of five measurements) was 89.8 ± 0.3% of the theoretical density, using the calculated theoretical density value from Table 4.

**Figure 3:**
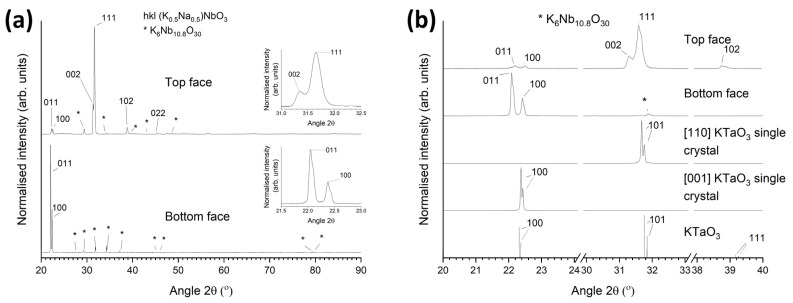
(**a**) XRD patterns of the as-sintered top and bottom faces of the 98.5 mol% (K_0.5_Na_0.5_)NbO_3_-1.5 mol% Ba_1.05_Nb_0.77_O_3_ sample from Figure 2: (**b**) XRD patterns of the samples in the range 20–40° 2θ, along with XRD patterns of [110] and [001]-oriented KTaO_3_ single-crystal substrates and the stick pattern for KTaO_3_.

XRD patterns of the as-sintered top and bottom faces of this sample are shown in Figure 3a. The insets show the major peaks. Both patterns were indexed using Crystallography Open Database pattern #96-230-0500 for (K_0.5_Na_0.5_)NbO_3_ (orthorhombic, *Amm2*). The pattern of the top face contains very strong 111 and 002 peaks and weaker 011, 100, 102, 022, and 202 peaks. The pattern of the bottom face contains strong 011 and 100 peaks and possibly very weak 022 and 200 peaks, visible if the intensity is viewed with a square-root scale. Both patterns contain peaks of a K_6_Nb_10.8_O_30_ second phase. For an X-ray diffractometer in the Bragg–Brentano configuration, only crystallographic planes which are parallel to the sample surface can diffract X-rays [62]. The appearance of very intense peaks shows that a large portion of the sample is single crystalline and that single crystals from a particular part of the sample grew with the same orientation. The weaker peaks from the top face of the sample may belong to single crystals that grew in a different orientation or to remaining matrix grains. The single crystals in the bottom part of the sample have grown in a different orientation to those in the top part of the sample. The splitting of the major peaks (111/002 for the top face and 011/100 for the bottom face) indicates the presence of non-180° ferroelectric or ferroelastic domains [63,64,65].

Figure 3b shows XRD patterns of the samples in the range 20–40° 2θ. XRD patterns of [110] and [001]-oriented KTaO_3_ single-crystal substrates scanned under the same conditions are also shown for comparison, along with the XRD stick pattern for KTaO_3_ (Crystallography Open Database pattern #96-210-2088 for KTaO_3_, cubic, $Pm\overset{-}{3}m$). FWHM values of the major peaks of the samples and single-crystal substrates were measured using the intermediate Lorentzian peak profile in Match! (Table 1). The Kα_2_ peaks were removed from the spectra before the FWHM values were measured. The peaks of the samples are much broader than the peaks of the KTaO_3_ substrates, and their corresponding FWHM values are larger. The increase in broadness and FWHM of the sample peaks compared to those of the KTaO_3_ substrates indicates the presence of strains, dislocations, ferroelastic domain walls, or local compositional variations in the KNBaN single crystals grown by seed-free SSCG [62,64,66,67,68].

**Figure 4:**
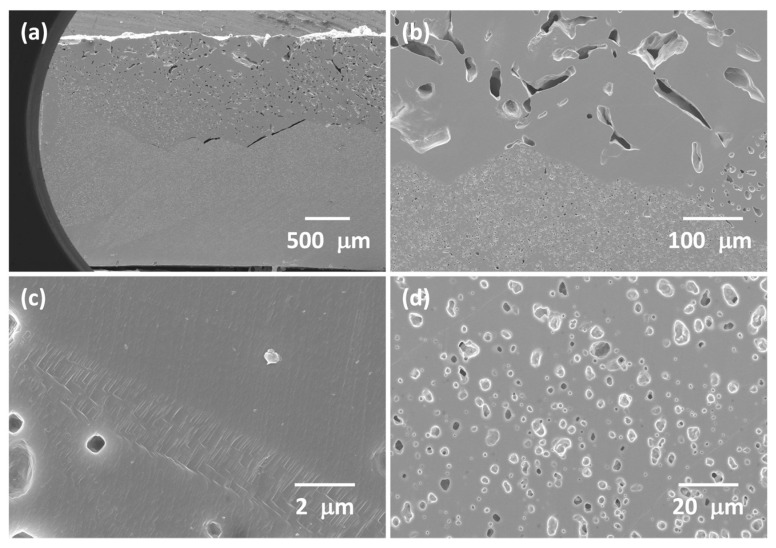
Cross-section SEM micrographs of a 98.5 mol% (K_0.5_Na_0.5_)NbO_3_-1.5 mol% Ba_1.05_Nb_0.77_O_3_ sample sintered at 1135 °C for 20 h: (**a**,**b**) single-crystal regions; (**c**) boundary between two single crystals; (**d**) porosity in the single crystal.

SEM micrographs of the polished and etched cross section of another KNBaN sample sintered at 1135 °C for 20 h are shown in Figure 4. Single crystals appear to have grown from the top and bottom faces of the sample. The sample is very porous. Estimates of the porosity measured from SEM micrographs are given in Table 2. The porosity values agree reasonably well with the density value of the sample in Figure 2. The single crystals that grew in the top part of the sample have fewer but larger pores than the single crystals that grew in the bottom part of the sample. The single crystals that grew in the top part of the sample are more porous than the single crystals that grew in the bottom part of the sample, and the porosity is less uniform. This probably explains the different colours of the single crystals in Figure 2 as the light will be reflected and scattered to different degrees by the differing number and size of the pores [69,70]. The boundaries between the crystals growing from the top and bottom parts of the sample are clearly faceted (Figure 4a,b). The boundary between two single crystals is shown in Figure 4c. The boundary does not look like a normal grain boundary, instead appearing as a series of steps. This boundary may possibly be a low-angle grain boundary, appearing as a series of dislocations [71]. Apart from this boundary dividing the top and bottom parts of the sample, no other grain boundaries were found. Porosity from a single crystal that grew in the bottom part of the sample is shown in Figure 4d. The pores are rectangular in shape and are aligned in the same direction. The EPMA results of the other half of the sample from Figure 4 are given in Table 3. The single crystals are slightly deficient in Na and K compared to the nominal composition. Ba_1.05_Nb_0.77_O_3_ has formed a complete solid solution with KNN.

**Figure 5:**
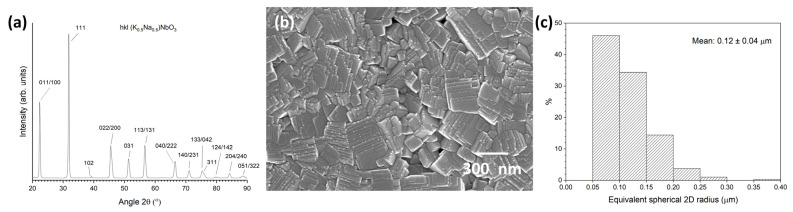
(**a**) XRD pattern of the as-sintered face of an unsuccessful 98.5 mol% (K_0.5_Na_0.5_)NbO_3_-1.5 mol% Ba_1.05_Nb_0.77_O_3_ sample sintered at 1135 °C for 20 h; (**b**) SEM micrograph of the sample; (**c**) grain-size distribution of the sample.

Figure 5a shows a bulk XRD pattern of the as-sintered face of a KNBaN sample sintered at 1135 °C for 20 h in which seed-free SSCG did not take place. The pattern is typical of a polycrystalline ceramic and is indexed using Crystallography Open Database pattern #96-230-0500 for (K_0.5_Na_0.5_)NbO_3_ (orthorhombic, *Amm2*). The peaks are broad, due to the submicron size of the grains [61]. Figure 5 also shows an SEM micrograph of the same sample (Figure 5b). The sample consists of cubic submicron grains with stepped surfaces. Many small dots are present on the steps, but they are too small to measure their composition with SEM-EDS. Figure 5c shows the grain-size distribution of the sample. The grain-size distribution is very narrow and unimodal, with a mean grain size and standard deviation of 0.12 ± 0.04 μm.

### 3.2. 98.5 mol% (K_0.5_Na_0.5_)NbO_3_-1.5 mol% Ba_1.05_Nb_0.77_O_3_ Seeded Solid-State Crystal Growth: [001] KTaO_3_ Seed

**Figure 6:**
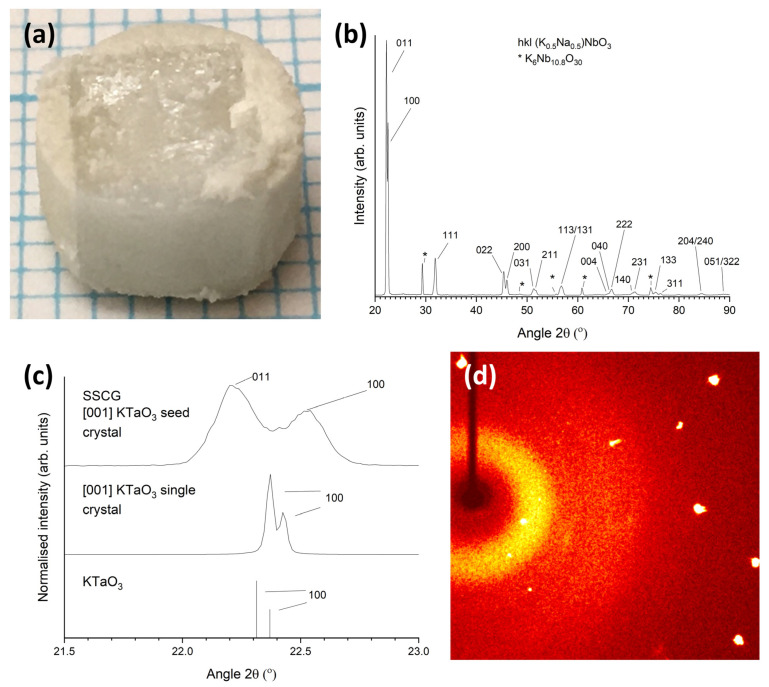
(**a**) Picture of a 98.5 mol% (K_0.5_Na_0.5_)NbO_3_-1.5 mol% Ba_1.05_Nb_0.77_O_3_ single crystal grown on a [001] KTaO_3_ seed crystal by sintering at 1135 °C for 20 h. Each blue line = 1 mm; (**b**) XRD pattern of the top face of the as-sintered sample in (**a**); (**c**) XRD pattern of the sample in (**a**) in the range 21.5–23.0° 2θ, along with an XRD pattern of the [001]-oriented KTaO_3_ single-crystal substrate and the stick pattern for KTaO_3_; (**d**) diffraction pattern of the 98.5 mol% (K_0.5_Na_0.5_)NbO_3_-1.5 mol% Ba_1.05_Nb_0.77_O_3_ single crystal taken by single-crystal XRD.

A picture of a KNBaN single crystal grown on a [001] KTaO_3_ seed crystal is shown in Figure 6a. A cube-shaped single crystal has grown in the sample. Polycrystalline regions can be seen at the edges of the sample. An XRD pattern of the as-sintered top face of this sample is shown in Figure 6b. The pattern was indexed using Crystallography Open Database pattern #96-230-0500 for (K_0.5_Na_0.5_)NbO_3_. Unlike the patterns in Figure 3, as well as strong peaks belonging to the single crystal (in this case, 011 and 100 peaks), there are many peaks belonging to the polycrystalline regions at the edges of the sample. Peaks belonging to a K_6_Nb_10.8_O_30_ second phase are also present. Figure 6c shows a magnified region of the XRD pattern in the range of 21.5–23.0° 2θ. The XRD pattern of the [001] KTaO_3_ single-crystal substrate and the Crystallography Open Database stick pattern #96-210-2088 for KTaO_3_ are also shown for reference. The KNBaN single crystal has grown epitaxially on the KTaO_3_ seed crystal. The d-spacing and FWHM of the 011 and 100 peaks of the single crystal are given in Table 1. It is clear that the peaks of the single crystal grown by SSCG on a [001] KTaO_3_ seed crystal have shifted to lower d-spacing values than the peaks of the single crystals grown by seed-free SSCG. The 011 and 100 peaks of the seeded KNN SSCG single crystal are also more closely-spaced than the 011 and 100 peaks of the seed-free KNN SSCG single crystal, indicating that epitaxial growth of the KNN single crystal on the KTaO_3_ seed crystal has caused a small change in lattice parameters. The FWHM values of the 011 and 100 peaks of the seeded KNN SSCG single crystal are larger than those of the 011 and 100 peaks of the seed-free KNN SSCG single crystal, indicating that epitaxial growth of the KNN single crystal on the KTaO_3_ seed crystal has caused an increase in strain and/or a change in domain structure.

Figure 6d shows a diffraction pattern of the KNBaN single crystal taken by single-crystal XRD. The appearance of Bragg peaks confirms that the sample is a single crystal [72]. The single-crystal XRD results for this sample are given in Table 4. The full results are given in Tables S1–S6 of the Supplementary Materials. The data is refined using a monoclinic unit cell with space group *P2*. KNN can be indexed with an orthorhombic or monoclinic unit cell depending on the choice of axes [73]. The theoretical density value in Table 4 is calculated using the nominal composition of KNBaN. The theoretical density is higher than that for KNN [74], due to the incorporation of Ba.

**Figure 7:**
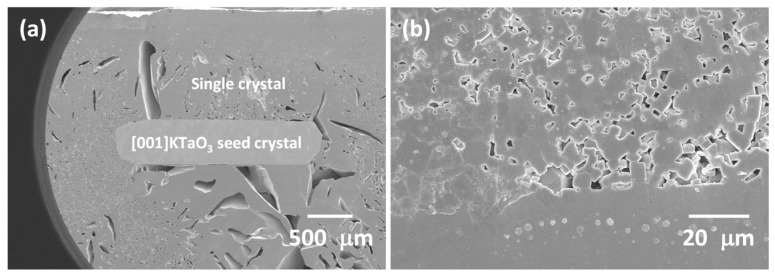
(**a**) SEM micrograph of a 98.5 mol% (K_0.5_Na_0.5_)NbO_3_-1.5 mol% Ba_1.05_Nb_0.77_O_3_ sample with [001] KTaO_3_ seed crystal sintered at 1135 °C for 5 h; (**b**) SEM micrograph of the matrix region in part (**a**).

Figure 7a shows a cross-sectional polished and etched SEM micrograph of a KNBaN sample with [001] KTaO_3_ seed crystal sintered at 1135 °C for 5 h. A single crystal has grown throughout almost the whole sample. The single crystal is very porous, with a wide range of pore sizes. The mean porosity value is intermediate between that of the KNBaN single crystals grown by seed-free SSCG (Table 2). A region of matrix grains is visible in the top left corner of Figure 7a. This region is shown in Figure 7b. The edge of the single crystal is visible at the bottom of the micrograph. Grain growth has taken place, with cubic grains of up to 10 μm in diameter present. EDS of the second-phase particles on the single crystal shows them to contain Na, K, and Cl. They are probably contamination picked up during sample polishing and etching.

### 3.3. 98.5 mol% (K_0.5_Na_0.5_)NbO_3_-1.5 mol% Ba(Cu_0.13_Nb_0.66_)O_3_ Seed-Free Solid-State Crystal Growth

**Figure 8:**
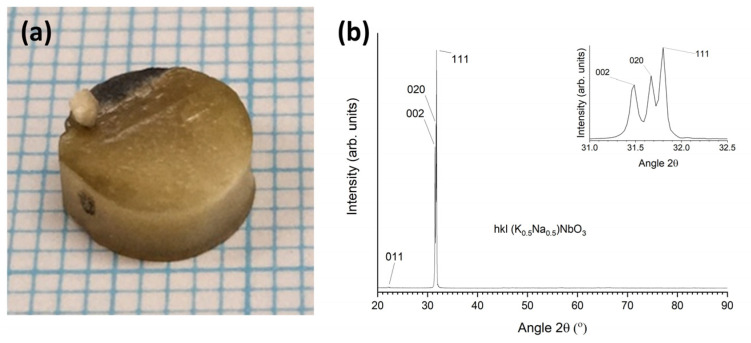
(**a**) Picture of a 98.5 mol% (K_0.5_Na_0.5_)NbO_3_-1.5 mol% Ba(Cu_0.13_Nb_0.66_)O_3_ sample sintered at 1125 °C for 21 h. Each blue line = 1 mm; (**b**) XRD pattern of a sectioned and polished 98.5 mol% (K_0.5_Na_0.5_)NbO_3_-1.5 mol% Ba(Cu_0.13_Nb_0.66_)O_3_ sample sintered at 1125 °C for 21 h.

A single crystal grown by seed-free SSCG in a KNBaCuN sample is shown in Figure 8a. A single crystal, bronze in colour, has grown through most of the sample. A polycrystalline region, grey in colour, is present in the top left part of the sample. The sample is similar in appearance to those grown by Ahn et al. [39,42]. The polycrystalline part of the sample was removed by grinding and the Archimedes density of the single-crystal part was measured as before. The Archimedes density (mean and standard deviation of five measurements) is 96.6 ± 0.3% theoretical density, using the theoretical density value from Table 4. The density is slightly lower than that of other KNN single crystals prepared by seed-free SSCG [54]. An XRD pattern of a sectioned and polished sample prepared under identical conditions is shown in Figure 8b. The pattern was indexed using Crystallography Open Database pattern #96-230-0500 for (K_0.5_Na_0.5_)NbO_3_. Strong 002, 020, and 111 peaks are present, as well as a weak 011 peak. A magnified plot of the intense peaks is shown in the inset. Very weak 022, 202, and 222 peaks, as well as very weak secondary-phase peaks of K_3_Nb_8_O_21_ or K_6_Nb_10.8_O_30_, are visible if the intensity is viewed with a log scale. Unlike the corresponding seed-free SSCG KNBaN sample (Figure 3, top face), the peaks at 31~32° 2θ are narrow and are split into three peaks. Peak positions and FWHM are given in Table 1. The FWHM values for the 111 and 020 peaks of the seed-free SSCG KNBaCuN sample are smaller than the corresponding values for the seed-free SSCG KNBaN sample, indicating less strain or a change in domain structure in the KNBaCuN single crystal. The XRD pattern for this sample was taken on a polished sample, whereas the pattern for the seed-free SSCG KNBaN sample was taken on the as-sintered sample. Possibly polishing has removed some of the strain from the sample.

**Figure 9:**
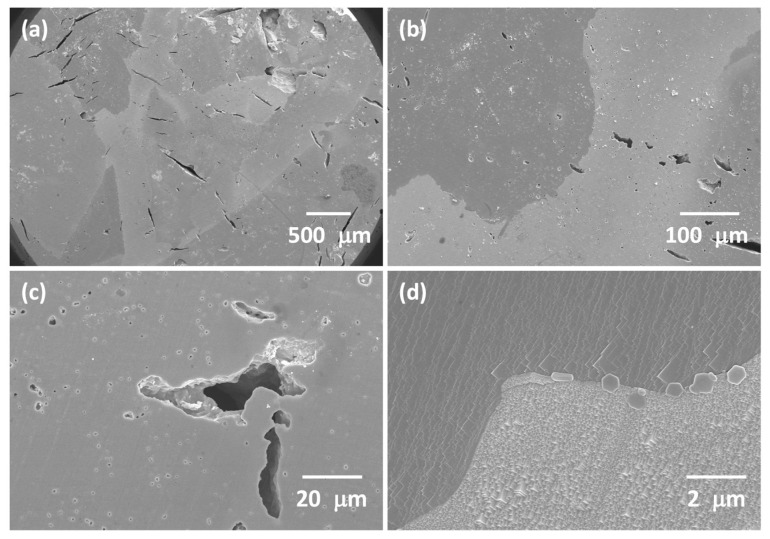
SEM micrographs of a 98.5 mol% (K_0.5_Na_0.5_)NbO_3_-1.5 mol% Ba(Cu_0.13_Nb_0.66_)O_3_ sample sintered at 1125 °C for 20 h: (**a**) single crystals; (**b**,**c**) porosity in the single crystals; (**d**) boundary between two single crystals.

SEM micrographs of a polished and etched KNBaCuN sample sintered at 1125 °C for 20 h are shown in Figure 9. This sample was polished on its top face. In this sample, several single crystals have grown (Figure 9a). This sample also contains pores (Figure 9b,c), although far fewer than the corresponding KNBaN sample. The porosity is approximately between half and one quarter of that of the corresponding KNBaN sample (Table 2). The porosity value agrees well with the theoretical density of the sample in Figure 8a. The grain boundaries between the single crystals appear to be regular grain boundaries and are faceted (Figure 9d). The steps on the surfaces of the single crystals in (Figure 9d) may be present because the surfaces are vicinal or may have been caused by the faceting of the polished surfaces into lower-energy crystallographic planes during thermal etching [75,76]. SEM-EDS of the hexagon-shaped phases in Figure 9d shows them to contain Si and Al. They are probably contamination picked up during sample polishing and etching.

Table 5 shows EPMA results for a KNBaCuN sample sintered at 1125 °C for 21 h. Similar to the KNBaN sample, this sample is also Na and K deficient. Most of the Ba(Cu_0.13_Nb_0.66_)O_3_ appears to have entered a solid solution with (K_0.5_Na_0.5_)NbO_3_. The Cu is assumed to enter the B-site of the perovskite lattice [77,78]. Some Cu may have not entered into a solid solution but the small amount of Cu makes accurate measurement difficult. The origin of the trace amount of Sb is not known, as this sample was buried in 98.5 mol% (K_0.5_Na_0.5_)NbO_3_-1.5 mol% Ba(Cu_0.13_Nb_0.66_)O_3_ packing powder during sintering.

### 3.4. 98.5 mol% (K_0.5_Na_0.5_)NbO_3_-1.5 mol% Ba(Cu_0.13_Nb_0.66_)O_3_ Seeded Solid-State Crystal Growth: [001] KTaO_3_ Seed

**Figure 10:**
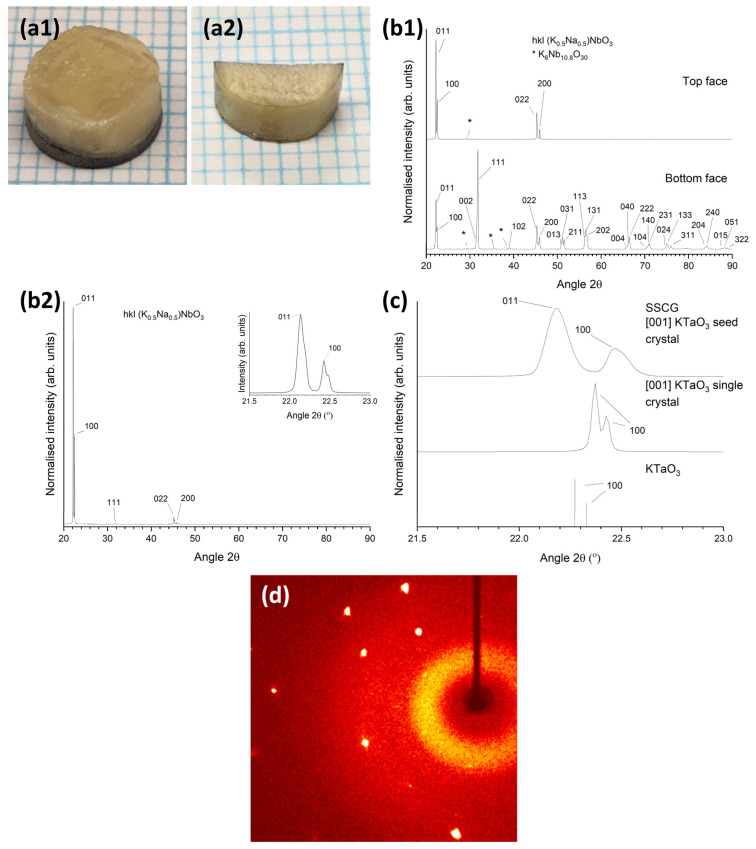
(**a1**,**a2**) Pictures of 98.5 mol% (K_0.5_Na_0.5_)NbO_3_-1.5 mol% Ba(Cu_0.13_Nb_0.66_)O_3_ single crystals grown on [001] KTaO_3_ seed crystals by sintering at 1125 °C for 10 h. Each blue line = 1 mm; (**b1**,**b2**) XRD patterns of the as-sintered samples. Patterns (**b1**,**b2**) correspond to (**a1**,**a2**) respectively; (**c**) XRD pattern of the sample in (**b1** top face) in the range 21.5–23.0° 2θ, along with the XRD pattern of the [001]-oriented KTaO_3_ single-crystal substrate and the stick pattern for KTaO_3_; (**d**) diffraction pattern of the 98.5 mol% (K_0.5_Na_0.5_)NbO_3_-1.5 mol% Ba(Cu_0.13_Nb_0.66_)O_3_ single crystal taken by single-crystal XRD.

Pictures of single crystals grown in KNBaCuN samples with [001] KTaO_3_ seed crystals sintered at 1125 °C for 10 h are shown in Figure 10. In Figure 10(a1) a single crystal has partially grown through the sample and in Figure 10(a2) a single crystal has grown throughout the whole sample (the sample was sectioned before the picture was taken). Figure 10b shows XRD patterns of the as-sintered samples in Figure 10a. All patterns were indexed using Crystallography Open Database pattern #96-230-0500 for (K_0.5_Na_0.5_)NbO_3_. The pattern of the top face of the sample in Figure 10(b1) shows strong *0kl* and *h00* peaks, along with very weak 104 and 033 peaks (visible when intensity is viewed with a log scale), indicating that the top part of the sample in Figure 10(a1) is a single crystal. The pattern of the bottom face shows that the bottom part of the sample is polycrystalline. A K_6_Nb_10.8_O_30_ second phase is also present. Figure 10(b2) shows a pattern of the top face of the sample from Figure 10(a2). The pattern was indexed using Crystallography Open Database pattern #96-230-0500 for (K_0.5_Na_0.5_)NbO_3_. Strong 011 and 100 peaks are present, as well as weak 111, 022, and 200 peaks. Very weak 031, 113, and 222 peaks are visible if the intensity is viewed with a log scale, along with a very-weak secondary-phase peak (K_6_Nb_10.8_O_30_). A magnified plot of the 011 and 100 peaks is shown in the inset.

Figure 10c shows the XRD pattern of the sample from (Figure 10(b1), top face) in the range 21.5–23.0° 2θ, along with the XRD pattern of the [001]-oriented KTaO_3_ single-crystal substrate and the stick pattern for KTaO_3_. The KNBaCuN single crystal has grown epitaxially on the KTaO_3_ seed crystal. The d-spacings and FWHM values of the 011 and 100 peaks of the KNN single crystal are given in Table 1. Similar to the KNBaN single crystal grown on a [001] KTaO_3_ seed crystal, there is a mismatch in d-spacing between the KNN single crystal and the [001] KTaO_3_ substrate. The mismatch between the KNN single crystal 100 peak and the 100 peak of the KTaO_3_ seed crystal is smaller for the KNBaCuN single crystal (0.0164 Å) than for the KNBaN single crystal (0.0261 Å). The FWHM values of the 011 and 100 peaks of the KNBaCuN single crystal are also smaller than those of the KNBaN single crystal grown on a [001] KTaO_3_ seed crystal, indicating less strain in the KNBaCuN single crystal. The 011 and 100 peaks of the KNBaCuN single crystal are more clearly separated than those of the KNBaN single crystal due to their narrower widths (Figure 6c). Figure 10d shows a diffraction pattern of the KNBaCuN single crystal taken by single-crystal XRD. The appearance of Bragg peaks again confirms that the sample is a single crystal. The single crystal XRD results for a KNBaCuN single crystal grown on a [001] KTaO_3_ seed crystal by sintering at 1125 °C for 10 h are given in Table 4. The full results are given in Tables S7–S12 of the Supplementary Materials. The theoretical density value in Table 4 is calculated using the nominal composition of KNBaCuN. The small change in density and unit-cell parameters is due to the incorporation of Cu.

**Figure 11:**
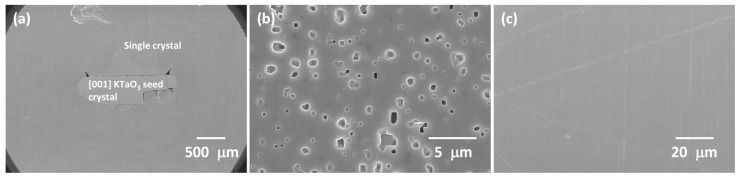
SEM micrographs of a 98.5 mol% (K_0.5_Na_0.5_)NbO_3_-1.5 mol% Ba(Cu_0.13_Nb_0.66_)O_3_ sample with [001] KTaO_3_ seed crystal sintered at 1125 °C for 10 h: (**a**) cross section of sample; (**b**) region near the seed crystal; (**c**) region near the sample edge.

Figure 11 shows polished and etched cross-sectional SEM micrographs of a KNBaCuN single crystal grown on a [001] KTaO_3_ seed crystal [the other half of the sample from Figure 10(a2)]. The single crystal is much less porous than the corresponding KNBaN single crystal. The region of the single crystal next to the seed crystal is shown in Figure 11b. The pores are rectangular in shape and are aligned in the same direction. Porosity in this region is approximately equal to that of the seed-free SSCG KNBaN single crystal grown from the bottom of the sample (Table 2). The region of the single crystal next to the sample edge is shown in Figure 11c. This region is completely pore free.

Table 6 shows the EPMA results for a KNBaCuN sample with a [001] KTaO_3_ seed crystal sintered at 1125 °C for 10 h. Similar to the previous samples, this sample is also Na and K deficient. Compared to the KNBaN sample, the KNBaCuN samples are more alkali deficient. All of the Ba(Cu_0.13_Nb_0.66_)O_3_ appears to have entered solid solution with KNN. The sample contains a trace amount of Sb. For this sample, a 98.5 mol% (K_0.5_Na_0.5_)(Nb_0.99_Sb_0.01_)O_3_-1.5 mol% Ba(Cu_0.13_Nb_0.66_)O_3_ packing powder was used during sintering. However, the Sb content is the same as that of the KNBaCuN sample prepared by seed-free SSCG with a (K_0.5_Na_0.5_)NbO_3_-1.5 mol% Ba(Cu_0.13_Nb_0.66_)O_3_ packing powder (Table 5). Use of the (K_0.5_Na_0.5_)(Nb_0.99_Sb_0.01_)O_3_-1.5 mol% Ba(Cu_0.13_Nb_0.66_)O_3_ packing powder did not appear to affect the composition of the KNBaCuN single crystal. 

**Figure 12:**
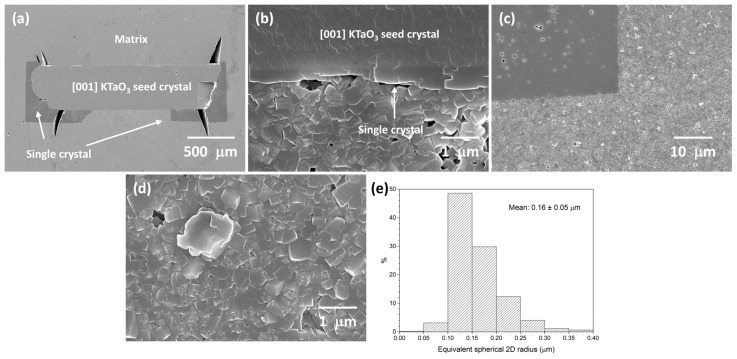
SEM micrographs of an unsuccessful 98.5 mol% (K_0.5_Na_0.5_)NbO_3_-1.5 mol% Ba(Cu_0.13_Nb_0.66_)O_3_ sample with [001] KTaO_3_ seed crystal sintered at 1125 °C for 10 h: (**a**) cross section of sample; (**b**) region near seed crystal; (**c**) porosity in single crystal; (**d**) matrix grains; (**e**) grain-size distribution of the matrix grains.

Figure 12 shows SEM micrographs of a KNBaCuN SSCG experiment that was unsuccessful. Single-crystal growth has only taken place at the edges and along part of the bottom face of the seed crystal (Figure 12a). For the rest of the bottom face of the seed crystal, single-crystal growth has barely taken place (Figure 12b). Even in the regions where single-crystal growth has taken place, it is very limited compared to the sample in Figure 11. The single crystal contains small pores (Figure 12c). Matrix grain size in this sample is submicron and no abnormal grain growth appears to have taken place (Figure 12d). The matrix is dense and contains small pores. Second-phase particles are visible in the matrix (Figure 12c,d). EDS shows these particles contain Na, K, Cu, and Nb and are Cu rich, containing between 17–27 at.% Cu. Figure 12e shows the grain-size distribution of the matrix grains. The grain-size distribution is narrow and unimodal, with a mean grain size and standard deviation of 0.16 ± 0.05 μm.

### 3.5. 98.5 mol% (K_0.5_Na_0.5_)NbO_3_-1.5 mol% Ba(Cu_0.13_Nb_0.66_)O_3_ Seeded Solid-State Crystal Growth: [110] KTaO_3_ Seed

**Figure 13:**
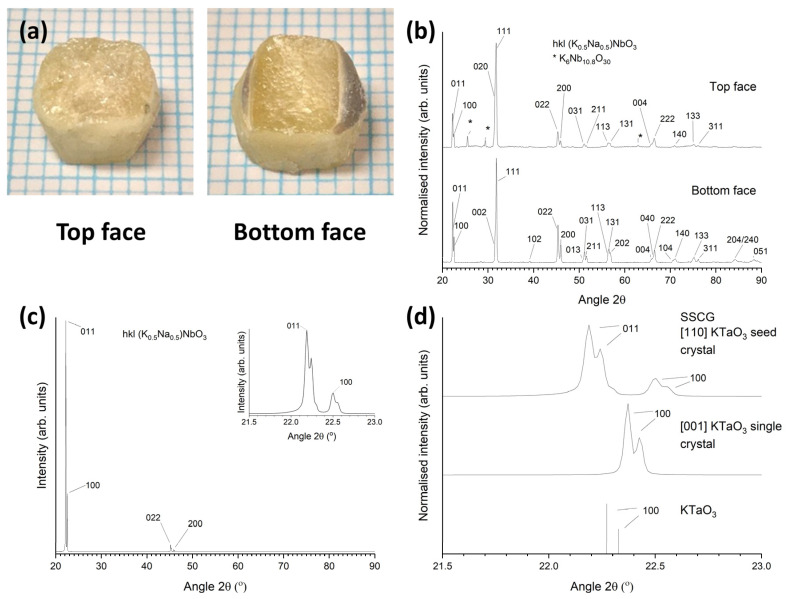
(**a**) Pictures of a 98.5 mol% (K_0.5_Na_0.5_)NbO_3_-1.5 mol% Ba(Cu_0.13_Nb_0.66_)O_3_ sample with [110] KTaO_3_ seed crystal sintered at 1125 °C for 10 h. Each blue line = 1 mm; (**b**) XRD patterns of the above sample as-sintered; (**c**) XRD pattern of the sample after vertical cross sectioning and polishing; (**d**) XRD pattern of the sample in (**c**) in the range 21.5–23.0° 2θ, along with the XRD pattern of the [001]-oriented KTaO_3_ single-crystal substrate and the stick pattern for KTaO_3_.

Figure 13a shows pictures of a KNBaCuN sample with [110] KTaO_3_ seed crystal sintered at 1125 °C for 10 h. A single crystal has grown throughout almost the whole sample. The bottom face of the sample shows some grey-coloured polycrystalline regions. Figure 13b shows bulk XRD patterns of the as-sintered top and bottom faces of this sample. Both patterns were indexed using Crystallography Open Database pattern #96-230-0500 for (K_0.5_Na_0.5_)NbO_3_. Unlike the sample grown with a [001] KTaO_3_ seed crystal, this sample appears to be polycrystalline on both faces. Figure 13c shows an XRD pattern of the cross-sectioned and polished face of the same sample. The pattern shows strong *0kl* and *h00* peaks. This shows that the sample is actually a single crystal. A magnified plot of the 011 and 100 peaks is shown in the inset.

Figure 13d shows the XRD pattern of the sample from Figure 13c in the range 21.5–23.0° 2θ, along with the XRD pattern of the [001]-oriented KTaO_3_ single-crystal substrate and the stick pattern for KTaO_3_. The d-spacings and FWHM values of the 011 and 100 peaks of the KNN single crystal are given in Table 1. The peaks are noticeably narrower and the values of FWHM are smaller than the corresponding peaks of the KNBaCuN sample grown on a [001] KTaO_3_ seed crystal (Figure 10(b2,c)). The FWHM values of the 011 and 100 peaks are similar to that of the [110] KTaO_3_ substrate. The XRD pattern in Figure 13c,d was taken using a polished sample whereas the XRD pattern in Figure 10(b2,c) was taken using the as-grown sample. Possibly polishing can remove some of the strain in the sample.

**Figure 14:**
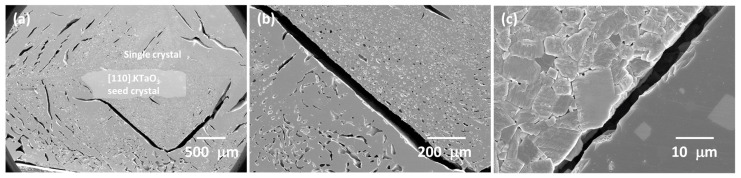
SEM micrographs of a 98.5 mol% (K_0.5_Na_0.5_)NbO_3_-1.5 mol% Ba(Cu_0.13_Nb_0.66_)O_3_ sample with [110] KTaO_3_ seed crystal sintered at 1125 °C for 10 h: (**a**) cross section of sample; (**b**) single-crystal region; (**c**) region near sample edge.

Figure 14 shows SEM micrographs of the cross-sectioned, polished, and thermally etched sample shown in Figure 13. A single crystal has grown on the [110] KTaO_3_ seed crystal and has consumed almost all the matrix grains in the sample. The single crystal is very porous, similar to the KNBaN single crystal grown on a [001] KTaO_3_ seed crystal (Figure 7 and Table 2). The pores appear aligned in certain directions (Figure 14b). The boundary between the single crystal and the remaining matrix grains is shown in Figure 14c. Grain growth has taken place in the matrix in this sample. Some island grains are also trapped inside the single crystal.

## 4. Discussion

Several workers have studied the conditions necessary for seed-free SSCG to take place in (K_0.5_Na_0.5_)NbO_3_. Ahn et al. found that the formation of a liquid phase (by addition of CuO) and compensation of Na loss during sintering by donor doping (Ba) promoted seed-free SSCG [39]. Rahman et al. found that seed-free SSCG (with or without the addition of CuO) took place only within a certain range of donor addition [41]. Jiang et al. also found that seed-free SSCG only took place within a certain range of LiBiO_3_ addition [40]. Morimoto et al. found that adjusting the alkali/niobium ratio and the addition of Bi_2_O_3_ could control the number of single crystals that grew in K/Na-deficient samples, with too much Bi_2_O_3_ addition preventing crystal growth [43,54]. Donor doping also affects grain-growth behaviour in BaTiO_3_ and SrTiO_3_, with abnormal grain growth taking place up to a certain amount of donor dopant addition while further addition suppresses grain growth [79,80].

**Figure 15:**
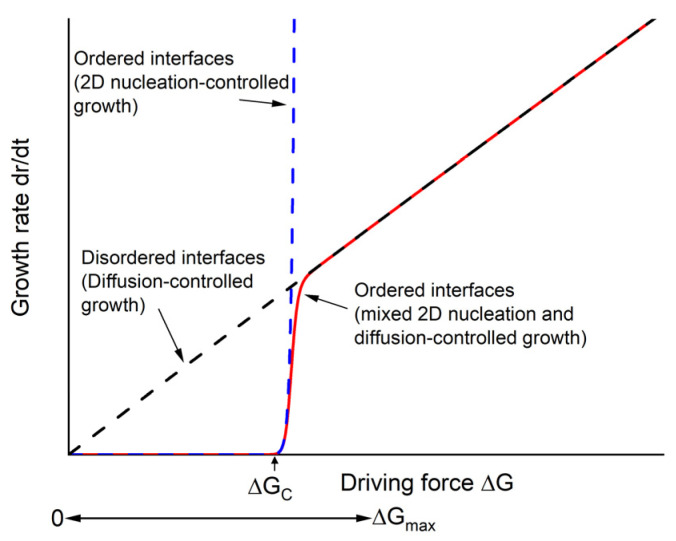
Schematic plot of growth rate vs. driving force for different types of interface (modified from [93]).

Solid-state crystal growth and seed-free solid-state crystal growth are basically a form of deliberately induced abnormal grain growth, in which some grains with sufficient driving force for growth grow to an unusually large size. The equation for ΔG, the driving force for the growth of a particular grain in a system with solid/liquid interfaces is [81,82,83]:

(1) $$∆G=2\gamma V_{m}\left(\frac{1}{\overset{-}{r}}-\frac{1}{r}\right)$$

where *γ* is the solid/liquid interfacial energy, *V_m_* is the molar volume, *r* is the radius of the grain and $\overset{-}{r}$ is the radius of a critical grain that has ΔG = 0, i.e., is neither growing nor shrinking (≈mean grain size). Numerous investigations have shown that abnormal grain-growth behaviour depends on the structure of the grain boundaries or solid/liquid interfaces [79,80,83,84,85,86,87,88,89,90,91,92]. Grain boundaries and solid/liquid interfaces can be disordered (rough) or ordered (faceted) on an atomic scale. If the solid–liquid interfaces are disordered, then atoms can easily attach at any point on the grain surface and grain growth is limited by the rate at which atoms can diffuse across the interface between the growing and shrinking grains. The grain-growth rate is a linear function of ΔG (the black dashed line in Figure 15 [93]) [81,94,95]. In this case, all grains with ΔG > 0 can grow and abnormal grain growth does not take place. If the solid–liquid interfaces are ordered, then atoms can only attach to the grain at low-energy sites such as 2D nuclei or steps formed by screw dislocations. The grain-growth rate is then a nonlinear function of ΔG [81,82,94,95]. The grain-growth rate for 2D nucleation-controlled growth is an exponential function of ΔG, as shown by the dashed blue curve in Figure 15. In this case, only grains with values of ΔG greater than the critical value ΔG_C_ are able to grow noticeably. For grains with ΔG > ΔG_C_, interface roughening takes place and the growth rate becomes diffusion-controlled (the solid red curve in Figure 15) [82,95,96]. Depending on the relative values of ΔG_C_ and ΔG_max_ (the driving force for the growth of the largest grain in the system), different types of non-normal growth such as pseudonormal (ΔG_C_ << ΔG_max_), abnormal (ΔG_C_ ≈ ΔG_max_), and stagnant (ΔG_C_ >> ΔG_max_) can take place [82,94,95]. Similar grain-growth behaviour takes place in systems with solid/solid grain boundaries [84,97,98]. This mechanism of describing grain-growth behaviour has been called the mixed control mechanism of grain growth [24,81,99].

In systems with ordered grain boundaries or solid–liquid interfaces, the edge-free energy *ε* (the excess energy caused by an edge such as the edge of a 2D nucleus or the edge of a step formed by a screw dislocation meeting the grain surface) plays an important role in governing the grain-growth behaviour. For systems with solid–liquid interfaces where grain growth is 2D nucleation-controlled, the relationship between ΔG_C_ and *ε* is [81,95,100,101]:

(2) $$∆G_{C}=\frac{\pi \varepsilon^{2}}{kTh}{\left(ln\psi n_{0}\right)}^{-1}$$

where *k* is the Boltzmann constant, *h* is the step height of the 2D nucleus and *n*_0_ is the number density of atoms in the liquid. $\psi =n^{*}\nu exp\left(\Delta G_{m}/kT\right)$, where n* is the number of atoms in a position near to a critical 2D nucleus, *ν* is the vibration frequency of atoms in the liquid, and Δ*G_m_* is the activation energy for jumping across the liquid–solid interface. The value of *ε*, and hence ΔG_C_, can be altered by changing the sintering atmosphere, material composition, and sintering temperature [76,81,84,85,95,102,103,104,105].

From Figure 5 and Figure 12, it can be seen that the (K_0.5_Na_0.5_)NbO_3_ grains in both KNBaN and KNBaCuN are cubic in shape, with sharp corners and edges. This implies a high value of *ε* and hence ΔG_C_ [92,94,106,107]. The submicron grain size, narrow grain-size distributions, and lack of abnormal grain growth even after extended sintering, i.e., stagnant grain growth, also imply high values of *ε* and ΔG_C_. Moon and Kang defined abnormal grains as grains with a size >3 times the mean grain size [108]. Following their definition, it appears that a small number of abnormal grains are just beginning to form in the KNBaN sample in Figure 5 after extended sintering, whereas abnormal grains did not yet form in the KNBaCuN sample in Figure 12. The occurrence of seed-free SSCG may then be due to stagnant grain growth followed by abnormal grain growth [81,82,94,104]. Initially, all the grains in the sample have ΔG < ΔG_C_ and can grow only very slowly. After some time, the largest grains in the system may grow large enough to have ΔG ≈ ΔG_C_. At this point, their growth rate increases rapidly (the solid red curve in Figure 15) and they form abnormal grains. As the other grains are barely growing, these abnormal grains can keep on growing to a large size by consuming all the matrix grains. Eventually, the abnormal grains impinge on each other and cannot grow further e.g., Figure 9. This is also seen in the work of Jiang et al. where there is a time delay before single-crystal growth takes place, and this time delay decreases as the sintering temperature increases [40]. The increased sintering temperature reduces *ε* [76,81,94,95] and hence ΔG_C_. This reduces the time needed for the largest grains in the sample to grow large enough to have ΔG ≈ ΔG_C_ and form abnormal grains.

In the case of seeded SSCG, the KTaO_3_ seed crystal acts as an artificial abnormal grain with ΔG > ΔG_C_. A KNN single crystal grows epitaxially on the seed crystal and consumes the surrounding matrix grains [19,34]. The growth behaviour of the single crystals grown on [001] KTaO_3_ seed crystals in the present work is very unusual. The [001] direction is the slowest growth direction in KNN, while the [110] direction is believed to be the fastest growth direction. Single crystals of KNN-based materials grown by SSCG on [001] KTaO_3_ seed crystals generally show limited growth (up to several hundred μm) and always show less growth compared to single crystals grown on [110] KTaO_3_ seed crystals [22,26,31]. However, in the present work, KNN single crystals grown on [001] KTaO_3_ seed crystals have grown to a size of several mm (Figure 6 and Figure 10(a1)), even consuming the entire pellet in one case (Figure 10(a2)). The growth rate of the single crystals is comparable to that of the crystal grown on a [110] KTaO_3_ seed crystal (Figure 13a). The driving force for the growth of the single crystal in SSCG is inversely proportional to the mean grain size and it was found that suppression of matrix grain growth could promote single-crystal growth of KNN in the [001] direction [109]. However, in other cases, single-crystal growth in the [001] direction was limited even for systems with micron-sized matrix grains [31,110]. So the reason for the unusually rapid growth in the [001] direction is not yet clear. The growth behaviour of the KNN single crystal on a [110] KTaO_3_ seed crystal is also noteworthy. Usually, the rapidly growing {110} planes grow themselves out of existence and the crystal takes a rhombohedral shape bounded by slowly growing {001} planes [22,26,27,31,32]. At this point, single-crystal growth essentially stops, which means that the size of the single crystal is limited by the size of the seed crystal. However, in the present work, the KNN single crystal had consumed almost the entire pellet (Figure 13a and Figure 14), implying that the single crystal keeps growing even when bounded by {001} planes.

The processing conditions, size, output ratio (defined as the% area of the top face of the sample face which was converted into single crystals), and chemical composition of the KNN single crystals grown in the present work are compared with those grown by seed-free SSCG in the literature (Table 7). The KNN single crystals grown in the present work are of comparable size to those grown by seed-free SSCG [33,39,40,41,42,43,46,54]. The processing temperatures and times are also similar to those in previous studies of seed-free SSCG. In some SSCG experiments in the present work, almost the entire volume of the pellet was converted into a single crystal (Figure 10 and Figure 13). This compares favourably with previous seed-free SSCG experiments in which multiple single crystals grow in a pellet, limiting their size [33,39,40,42,43,45,54], although Morimoto et al. could restrict the number of single crystals to one per pellet by careful control of the composition and dopant addition [43,54].

The single crystals are all alkali deficient (Table 3, Table 5 and Table 6) due to the evaporation of the volatile alkali elements during sintering. This is sometimes seen in (K_0.5_Na_0.5_)NbO_3_-based single crystals grown by SSCG [19,32], as well as in (K_x_Na_1-x_)NbO_3_ single crystals grown by other methods [111,112,113]. Charge neutrality is maintained through the formation of oxygen vacancies according to the defect reaction [114]:

(3) $$2A_{A}^{x}+O_{O}^{x}⇌2V_{A}^{\prime }+V_{O}^{●●}+2A\left(g\right)+\frac{1}{2}O_{2}\left(g\right)\;\;\;\left(A=K,\;\mathrm{Na}\right)$$

Control of alkali loss is important as a decrease in the alkali/niobium ratio affects the single-crystal growth behaviour through the formation of compensating oxygen vacancies [43,54]. Oxygen vacancies can also affect electrical properties [114]. The purity of the KNN single crystals grown in the present work is similar to that of KNN crystals prepared by other workers (Table 7). The chemical compositions of the KNBaCuN single crystals are similar to those grown by Ahn et al. [42]. They found that their single crystals contained less Na than K, but in the present work, the single crystals usually contained less K than Na. This may be due to the higher vapour pressure of K over KNN compared to Na [115] and the difficulty of accurately measuring alkali content using energy dispersive spectroscopy [116] as opposed to the wavelength-dispersive spectroscopy used in the present work. Compared to the single crystals grown by Morimoto et al., the single crystals in the present work are Na deficient [54]. Single crystals grown by Yao et al. [33] and Jiang et al. [40] were slightly Na excess and K deficient The samples in the present work are Na deficient in comparison. Alkali loss may possibly be reduced by using alkali-excess packing powder or by sealing the crucible lid with alumina cement. According to the EPMA results, all or almost all of the Ba_1.05_Nb_0.77_O_3_ and Ba(Cu_0.13_Nb_0.66_)O_3_ components enter a solid solution with (K_0.5_Na_0.5_)NbO_3_. However, the appearance of Cu-rich second-phase particles indicates otherwise for Ba(Cu_0.13_Nb_0.66_)O_3_ (Figure 12).

The KNBaN single crystals grown by seed-free SSCG in the present work grow in 011/100 orientation (with intense diffraction peaks at ~22° 2θ) or in 002/111 orientation (with intense diffraction peaks at ~32° 2θ) (Figure 3). KNN single crystals grown by seed-free SSCG by other workers grew with (100)_pseudocubic_ orientation with an intense diffraction peak at ~22° 2θ [39,40,42,52,53] or with (020)_orthorhombic_ orientation with an intense diffraction peak at ~32° 2θ [33,40]. The KNBaN single crystals grown by seed-free SSCG in the present work grow in the same orientations as those grown by other workers, the differences in Miller indices being due to the different unit cells used by different authors. The orientation of KNBaN and KNBaCuN single crystals grown on [001] KTaO_3_ seed crystals is controlled by the seed crystal as expected.

The single crystals grown in the KNBaN samples are very porous (Table 2, Figure 4 and Figure 7). During sintering, if pores are unable to migrate along with the single crystal/matrix grain boundary, they can separate from the boundary and become trapped in the single crystal [117,118,119]. Once trapped inside the crystal, the pores are very difficult to remove as the gas inside the pore must diffuse through the crystal lattice to the single crystal/matrix grain boundary [117]. Pore coalescence and swelling can also take place [120,121,122]. The single crystals grown in the KNBaCuN samples are less porous than the single crystals grown in the KNBaN samples (Table 2, Figure 9 and Figure 11), with the exception of the single crystal grown on the [110] KTaO_3_ seed crystal (Figure 14). The Archimedes density measurements of the seed-free SSCG samples also show that the KNBaCuN sample has a higher density than the KNBaN sample. The Ba(Cu_0.13_Nb_0.66_)O_3_ component is believed to form a liquid phase during sintering, which may help densify the sample before single-crystal growth begins [39,42]. CuO and Cu-containing compounds have been found to be effective sintering aids for (K_0.5_Na_0.5_)NbO_3_ [123,124]. This liquid phase may be the cause of the secondary-phase particles visible in Figure 12c,d).

The single crystals grown by seed-free SSCG in the KNBaCuN samples have fewer fine pores than the single crystal grown on the [001] KTaO_3_ seed crystal (Figure 9c and Figure 11b), although more larger pores are present. In the seeded SSCG sample, a large grain with ΔG > ΔG_C_ is already present in the form of the KTaO_3_ seed crystal. Therefore single-crystal growth probably takes place more quickly in the seeded SSCG sample than in the seed-free SSCG sample, where time is needed for a grain to grow large enough to have ΔG > ΔG_C_. This gives the seed-free SSCG sample more time to densify before single-crystal growth starts. In the seeded SSCG sample, crystal growth begins before the sample has fully densified. This also explains why the single-crystal region near the [001] KTaO_3_ seed crystal (Figure 11b) is porous while the region near the edge is dense (Figure 11c); the edges of the sample had enough time to completely densify before the single crystal reached them. The KNBaCuN single crystal grown on the [110] KTaO_3_ seed crystal may be more porous because the crystal is expected to grow more rapidly than the crystal grown on the [001] KTaO_3_ seed crystal. The crystal grows and incorporates the pores before they can be removed from the matrix. The smaller pores in the single crystals are cubic in shape and align along certain directions. The pores act as ”negative crystals” [117] and try to take on the equilibrium crystal shape [125].

Single-crystal growth of (K_0.5_Na_0.5_)NbO_3_-based single crystals by SSCG has generally suffered from three problems: the need for expensive KTaO_3_ seed crystals, porosity in the single crystals and the limited size of crystals that can be grown. The present work has shown that the addition of Ba(Cu_0.13_Nb_0.66_)O_3_ to (K_0.5_Na_0.5_)NbO_3_ is effective in reducing porosity in the single crystals and increasing the size of the grown crystals, particularly in the [001] growth direction. This is particularly useful as the [001] orientation has the best piezoelectric properties [12,35,36]. The reason why some single-crystal growth experiments are unsuccessful is not yet known. As mentioned earlier, both powders are free of secondary phases even after storage (Figure 1) so there does not appear to be a problem with deterioration of the powders. In addition, the XRD patterns of successful growth experiments show second-phase peaks, so the presence of a second phase does not appear to prevent single-crystal growth. In the seed-free SSCG experiments, a sample may by chance fail to have any grains large enough to grow into single crystals, but the seeded SSCG experiments always have at least one grain that is large enough to grow i.e., the seed crystal. Further experiments need to be carried out to address this problem.

## 5. Conclusions

Single crystals of (K_0.5_Na_0.5_)NbO_3_-Ba_1.05_Nb_0.77_O_3_ and (K_0.5_Na_0.5_)NbO_3_- Ba(Cu_0.13_Nb_0.66_)O_3_ were grown by seed-free solid-state crystal growth and seeded solid-state crystal growth. Single crystals several millimetres in size were grown using [001]-oriented KTaO_3_ seed crystals in both systems. Such crystals are unusually large, as the [001] direction is the slowest growth direction in (K_0.5_Na_0.5_)NbO_3_, and are larger than (K_0.5_Na_0.5_)NbO_3_-based single crystals previously grown by this method. In the (K_0.5_Na_0.5_)NbO_3_-Ba_1.05_Nb_0.77_O_3_ system, single crystals grown by seed-free solid-state crystal growth and single crystals grown in the [001] direction using seeded solid-state crystal growth were very porous. In the (K_0.5_Na_0.5_)NbO_3_- Ba(Cu_0.13_Nb_0.66_)O_3_ system, porosity could be significantly reduced in single crystals grown by seed-free solid-state crystal growth and in single crystals grown in the [001] direction using seeded solid-state crystal growth. Single crystals grown in the [110] direction were still very porous despite the use of Ba(Cu_0.13_Nb_0.66_)O_3_. The combination of [001]-oriented KTaO_3_ seed crystals and Ba(Cu_0.13_Nb_0.66_)O_3_ addition allows large and dense single crystals to be grown, but the issue of repeatability still needs to be solved.

## Figures and Tables

**Table 1:** d-spacing and FWHM values of the major peaks of the 98.5 mol% (K_0.5_Na_0.5_)NbO_3_-1.5 mol% Ba_1.05_Nb_0.77_O_3_ (KNBaN) and 98.5 mol% (K_0.5_Na_0.5_)NbO_3_-1.5 mol% Ba(Cu_0.13_Nb_0.66_)O_3_ (KNBaCuN) single crystals and KTaO_3_ single-crystal substrates.

Peak	d-Spacing (Å)	FWHM (° 2θ)
KNBaN seed-free SSCG (top face) 111	2.8264	0.1541
KNBaN seed-free SSCG (top face) 002	2.8523	0.1327
KNBaN seed-free SSCG (bottom face) 011	4.0283	0.0517
KNBaN seed-free SSCG (bottom face) 100	3.9710	0.0657
KNBaN SSCG [100] KTaO_3_ seed crystal 011	3.9992	0.1687
KNBaN SSCG [100] KTaO_3_ seed crystal 100	3.945	0.1701
KNBaCuN seed-free SSCG 111	2.8096	0.0678
KNBaCuN seed-free SSCG 020	2.8203	0.0721
KNBaCuN seed-free SSCG 002	2.8368	0.1031
KNBaCuN SSCG [100] KTaO_3_ seed crystal 011	4.0067	0.0942
KNBaCuN SSCG [100] KTaO_3_ seed crystal 100	3.9547	0.0763
KNBaCuN SSCG [110] KTaO_3_ seed crystal 011	3.9945	0.0444
KNBaCuN SSCG [110] KTaO_3_ seed crystal 100	3.9404	0.0477
KTaO_3_ substrate 100	3.9711	0.0348
KTaO_3_ substrate 110	2.8227	0.0520

**Table 2:** Porosity values of the 98.5 mol% (K_0.5_Na_0.5_)NbO_3_-1.5 mol% Ba_1.05_Nb_0.77_O_3_ (KNBaN) and 98.5 mol% (K_0.5_Na_0.5_)NbO_3_-1.5 mol% Ba(Cu_0.13_Nb_0.66_)O_3_ (KNBaCuN) single crystals estimated from SEM micrographs.

Sample	Mean Porosity (%)	Standard Deviation (%)	Number of Micrographs Measured
KNBaN seed-free SSCG (top face)	12.91	9.73	5
KNBaN seed-free SSCG (bottom face)	7.41	2.24	3
KNBaN seeded SSCG [100] KTaO_3_ seed crystal	10.15	5.25	3
KNBaCuN seed-free SSCG	2.98	3.85	4
KNBaCuN seeded SSCG [100] KTaO_3_ seed crystal (region next to seed crystal)	8.32	1.17	2
KNBaCuN seeded SSCG [110] KTaO_3_ seed crystal	12.10	5.80	2

**Table 3:** EPMA results of a 98.5 mol% (K_0.5_Na_0.5_)NbO_3_-1.5 mol% Ba_1.05_Nb_0.77_O_3_ sample sintered at 1135 °C for 20 h. Results are normalised to 0.997 formula units of Nb. The nominal composition assumes that Ba_1.05_Nb_0.77_O_3_ forms a complete solid solution with (K_0.5_Na_0.5_)NbO_3_.

Element	Na	K	Ba	Nb
Top half	0.477 ± 0.007	0.472 ± 0.006	0.015 ± 0.000	0.997
Bottom half	0.483 ± 0.003	0.464 ± 0.005	0.016 ± 0.001	0.997
Nominal	0.493	0.493	0.016	0.997

**Table 4:** Single crystal XRD results of a 98.5 mol% (K_0.5_Na_0.5_)NbO_3_-1.5 mol% Ba_1.05_Nb_0.77_O_3_ (KNBaN) single crystal grown on a [001] KTaO_3_ seed crystal by sintering at 1135 °C for 20 h and a 98.5 mol% (K_0.5_Na_0.5_)NbO_3_-1.5 mol% Ba(Cu_0.13_Nb_0.66_)O_3_ (KNBaCuN) single crystal grown on a [001] KTaO_3_ seed crystal by sintering at 1125 °C for 10 h.

Sample	Crystal System	Space Group	Unit Cell Parameters (Å)	Unit Cell Angles (°)	Unit Cell Volume (Å^3^)	Formula Units per Unit Cell	Theoretical Density (g⋅ cm^−3^)	R_1_	wR_2_
KNBaN seeded SSCG [100] KTaO_3_ seed crystal	monoclinic	*P2*	a = 3.9761(4) b = 3.9637(5) c = 3.9918(4)	α = 90 β = 90.072(6) γ = 90	62.911(12)	1	4.58	0.0212	0.0575
KNBaCuN seeded SSCG [100] KTaO_3_ seed crystal	monoclinic	*P2*	a = 3.9714(6) b = 3.9683(5) c = 3.9996(6)	α = 90 β = 89.999(9) γ = 90	63.033(16)	1	4.56	0.0265	0.0715

**Table 5:** EPMA results of a 98.5 mol% (K_0.5_Na_0.5_)NbO_3_-1.5 mol% Ba(Cu_0.13_Nb_0.66_)O_3_ sample sintered at 1125 °C for 21 h. Results are normalised to 0.997 formula units of B-site cations. Cu is assumed to enter the B-sites. The nominal composition assumes that Ba(Cu_0.13_Nb_0.66_)O_3_ forms a complete solid solution with (K_0.5_Na_0.5_)NbO_3_.

Element	Na	K	Ba	Cu	Sb	Nb
	0.434 ± 0.014	0.416 ± 0.004	0.017 ± 0.001	0.001 ± 0.001	0.004 ± 0.000	0.992 ± 0.001
Nominal	0.493	0.493	0.015	0.002	0	0.995

**Table 6:** EPMA results of a 98.5 mol% (K_0.5_Na_0.5_)NbO_3_-1.5 mol% Ba(Cu_0.13_Nb_0.66_)O_3_ sample with [001] KTaO_3_ seed crystal sintered at 1125 °C for 10 h. Results are normalised to 0.997 formula units of B-site cations. Cu is assumed to enter the B-sites. The nominal composition assumes that Ba(Cu_0.13_Nb_0.66_)O_3_ forms a complete solid solution with (K_0.5_Na_0.5_)NbO_3_.

Element	Na	K	Ba	Cu	Sb	Nb
	0.413 ± 0.012	0.464 ± 0.004	0.015 ± 0.001	0.002 ± 0.001	0.004 ± 0.000	0.991 ± 0.001
Nominal	0.493	0.493	0.015	0.002	0	0.995

**Table 7:** Comparison of processing conditions, size, output ratio, and chemical composition of the 98.5 mol% (K_0.5_Na_0.5_)NbO_3_-1.5 mol% Ba_1.05_Nb_0.77_O_3_ (KNBaN) and 98.5 mol% (K_0.5_Na_0.5_)NbO_3_-1.5 mol% Ba(Cu_0.13_Nb_0.66_)O_3_ (KNBaCuN) single crystals grown in the present work with those grown by seed-free SSCG in the literature.

Sample Name	Nominal Composition	Composition	Sintering Temperature (°C)	Sintering Time (h)	Size (mm)	Output Ratio (Area%)	Reference
KNBaN seed-free SSCG	(K_0.493_Na_0.493_Ba_0.016_) Nb_0.997_O_3_	(K_0.472_Na_0.477_Ba_0.015_) Nb_0.997_O_3_/ (K_0.464_Na_0.483_Ba_0.016_) Nb_0.997_O_3_	1135	20–21	~5	~100	This work
KNBaN SSCG [100] KTaO_3_ seed crystal	(K_0.493_Na_0.493_Ba_0.016_) Nb_0.997_O_3_	Not analysed	1135	5–20 h	~5	~67	This work
KNBaCuN seed-free SSCG	(K_0.493_Na_0.493_Ba_0.015_) (Nb_0.995_Cu_0.002_)O_3_	(K_0.416_Na_0.434_Ba_0.017_) (Nb_0.992_Cu_0.001_Sb_0.004_)O_3_	1125	20–21	~7	~91	This work
KNBaCuN SSCG [100] KTaO_3_ seed crystal	(K_0.493_Na_0.493_Ba_0.015_) (Nb_0.995_Cu_0.002_)O_3_	(K_0.464_Na_0.413_Ba_0.015_) (Nb_0.991_Cu_0.002_Sb_0.004_)O_3_	1125	10	~7	~100	This work
KNBaCuN SSCG [110] KTaO_3_ seed crystal	(K_0.493_Na_0.493_Ba_0.015_) (Nb_0.995_Cu_0.002_)O_3_	Not analysed	1125	10	~8	~78	This work
KNBaCuN seed-free SSCG	(K_0.493_Na_0.493_Ba_0.015_) (Nb_0.995_Cu_0.005_)O_3_	(K_0.454_Na_0.426_Ba_0.014_) (Nb_1.106_Cu_0.000_)O_3_	1120	2	~23	~62–89	[42]
KNN-LiBiO_3_	99.5(99.6K_0.5_Na_0.5_NbO_3_– 0.4LiBiO_3_)–0.5MnO_2_	Na_0.525_K_0.443_NbO_3_	1105	21	~18	~93–99	[33]
KNN-LiBiO_3_	(1-x)Na_0.5_K_0.5_NbO_3_-xLiBiO_3_ (x = 0.001–0.006)	(Na_0.51_K_0.47_Bi_0.005_)Nb_1.125_O_3_ (1110 °C, 12 h)	1080–1120	3–24	~12	~90–100	[40]
KNN-CuO-Bi_2_O_3_	Na_0.495_K_0.495_NbO_3_-1.5 wt% CuO-0.5 wt% Bi_2_O_3_	(Na_0.494_K_0.468_Bi_0.004_) (Nb_1.032_Cu_0.002_)O_3_	1060	15	~15	~68	[54]

## Data Availability

The data presented in this study are available on request from the corresponding author.

## Supplementary Materials

The following supporting information can be downloaded at: https://www.mdpi.com/article/10.3390/ma16103638/s1, Table S1: Crystal data and structure refinement for a 98.5 mol% (K_0.5_Na_0.5_)NbO_3_-1.5 mol% Ba_1.05_Nb_0.77_O_3_ single crystal grown on a [001] KTaO_3_ seed crystal by sintering at 1135 °C for 20 h (KNBaN); Table S2: Fractional Atomic Coordinates (×10^4^) and Equivalent Isotropic Displacement Parameters (Å^2^ × 10^3^) for KNBaN. U_eq_ is defined as 1/3 of the trace of the orthogonalised U_IJ_ tensor; Table S3: Anisotropic Displacement Parameters (Å^2^ × 10^3^) for KNBaN. The Anisotropic displacement factor exponent takes the form: −2π^2^[h^2^a*^2^U_11_ + 2hka*b*U_12_ + …]; Table S4: Bond Lengths for KNBaN; Table S5: Bond Angles for KNBaN; Table S6: Atomic Occupancy for KNBaN; Table S7: Crystal data and structure refinement for a 98.5 mol% (K_0.5_Na_0.5_)NbO_3_-1.5 mol% Ba(Cu_0.13_Nb_0.66_)O_3_ single crystal grown on a [001] KTaO_3_ seed crystal by sintering at 1125 °C for 10 h (KNBaCuN); Table S8: Fractional Atomic Coordinates (×10^4^) and Equivalent Isotropic Displacement Parameters (Å^2^ × 10^3^) for KNBaCuN. U_eq_ is defined as 1/3 of the trace of the orthogonalised U_IJ_ tensor; Table S9: Anisotropic Displacement Parameters (Å^2^ × 10^3^) for KNBaCuN. The Anisotropic displacement factor exponent takes the form: −2π^2^[h^2^a*^2^U_11_ + 2hka*b*U_12_ + …]; Table S10: Bond Lengths for KNBaCuN; Table S11: Bond Angles for KNBaCuN; Table S12 Atomic Occupancy for KNBaCuN.
