# Supplementary material for: Comparison of (K0.5Na0.5)NbO3 Single Crystals Grown by Seed-Free and Seeded Solid-State Single Crystal Growth
Source: Materials (Basel). 2023 May 10;16(10):3638. doi: 10.3390/ma16103638 (PMC10224234; doi:10.3390/ma16103638)
Supplement: Supplementary file 1 [file materials-16-03638-s001.zip › materials-2290173-supplementary.pdf]

Table S1 Crystal data and structure refinement for a 98.5 mol % ($(K_{0.5}Na_{0.5})NbO_3$-1.5 mol % $Ba_{1.05}Nb_{0.77}O_3$ single crystal grown on a [001] $KTaO_3$ seed crystal by sintering at 1135 °C for 20 h (KNBaN).

Identification code	KNBaN
**Empirical formula**	$K_{0.5}Na_{0.5}NbO_3$
**Formula weight**	171.96
**Temperature/K**	296.15
**Crystal system**	monoclinic
**Space group**	P2
**a/Å**	3.9761(4)
**b/Å**	3.9637(5)
**c/Å**	3.9918(4)
**α/°**	90
**β/°**	90.072(6)
**γ/°**	90
**Volume/Å^3**	62.911(12)
**Z**	1
**ϱ_{calc}g/cm^3**	4.539
**μ/mm^{-1}**	5.421
**F(000)**	80.0
**Crystal size/mm^3**	0.2 × 0.1 × 0.05
**Radiation**	MoKα (λ = 0.71073)
**2Θ range for data collection/°**	10.214 to 52.862
**Index ranges**	$-4 \le h \le 4, -4 \le k \le 4, -5 \le l \le 4$
**Reflections collected**	633
**Independent reflections**	229 [R_{int} = 0.0160, R_{sigma} = 0.0146]
**Data/restraints/parameters**	229/1/26
**Goodness-of-fit on F^2**	1.006
**Final R indexes [I>=2σ (I)]**	R_1 = 0.0212, wR_2 = 0.0575
**Final R indexes [all data]**	R_1 = 0.0212, wR_2 = 0.0575
**Largest diff. peak/hole / e Å^{-3}**	0.86/-0.83
**Flack parameter**	0.48(13)

Table S2 Fractional Atomic Coordinates (×10^4) and Equivalent Isotropic Displacement Parameters (Å^2×10^3) for KNBaN. U_{eq} is defined as 1/3 of the trace of the orthogonalised U_{IJ} tensor.

Atom	x	y	z	U(eq)
Nb1	0	6192.8(2)	5000	4.6(5)
O1	0	11380(170)	5000	30(4)
O2	5000	6400(200)	5000	29(6)
O3	0	6360(190)	0	28(4)
K1	5000	11450(170)	0	13(6)
Na1	5000	11450(170)	0	13(6)

Table S3 Anisotropic Displacement Parameters (Å^2×10^3) for KNBaN. The Anisotropic displacement factor exponent takes the form: $-2\pi^2[h^2a^{*2}U_{11}+2hka^*b^*U_{12}+\ldots]$.

Atom	U_{11}	U_{22}	U_{33}	U_{23}	U_{13}	U_{12}
Nb1	4.4(5)	7.0(15)	2.4(5)	0	-0.2(3)	0
O1	39(5)	4(13)	46(6)	0	-14(4)	0
O2	30(5)	10(17)	46(6)	0	-10(4)	0
O3	35(5)	16(13)	35(5)	0	-10(4)	0
K1	17.7(15)	5(17)	16.3(16)	0	-2.1(11)	0
Na1	17.7(15)	5(17)	16.3(16)	0	-2.1(11)	0

Table S4 Bond Lengths for KNBaN.

Atom	Atom	Length/Å	Atom	Atom	Length/Å
Nb1	O1^1	1.91(7)	O2	K1^5	2.80(10)
Nb1	O1	2.06(7)	O2	K1	2.82(10)
Nb1	O2	1.990(4)	O2	Na1^3	2.82(10)
Nb1	O2^2	1.990(4)	O2	Na1^5	2.80(10)
Nb1	O3^3	1.997(3)	O2	Na1^1	2.80(10)
Nb1	O3	1.997(3)	O3	Nb1^{10}	1.997(3)
Nb1	K1^4	3.39(4)	O3	K1	2.83(9)
Nb1	K1^5	3.39(4)	O3	K1^2	2.83(9)
Nb1	Na1^4	3.39(4)	O3	K1^4	2.78(9)
Nb1	Na1^6	3.39(4)	O3	K1^1	2.78(9)
Nb1	Na1^5	3.39(4)	O3	Na1^2	2.83(9)
Nb1	Na1^1	3.39(4)	O3	Na1^4	2.78(9)
O1	Nb1^7	1.91(7)	O3	Na1^1	2.78(9)
O1	K1	2.8190(14)	K1	O1^{11}	2.8190(13)
O1	K1^8	2.8190(14)	K1	O1^{10}	2.8154(13)
O1	K1^2	2.8154(14)	K1	O1^9	2.8154(13)
O1	K1^3	2.8154(14)	K1	O2^{10}	2.82(10)
O1	Na1^2	2.8154(14)	K1	O2^7	2.80(10)
O1	Na1^8	2.8190(14)	K1	O2^{12}	2.80(10)
O1	Na1^3	2.8154(14)	K1	O3^{13}	2.78(9)
O2	Nb1^9	1.990(4)	K1	O3^7	2.78(9)
O2	K1^3	2.82(10)	K1	O3^9	2.83(9)
O2	K1^1	2.80(10)			

^{1}+X,-1+Y,+Z; ^{2}-1+X,+Y,+Z; ^{3}+X,+Y,1+Z; ^{4}-1+X,-1+Y,+Z; ^{5}+X,-1+Y,1+Z; ^{6}-1+X,-1+Y,1+Z; ^{7}+X,1+Y,+Z; ^{8}-1+X,+Y,1+Z; ^{9}1+X,+Y,+Z; ^{10}+X,+Y,-1+Z; ^{11}1+X,+Y,-1+Z; ^{12}+X,1+Y,-1+Z; ^{13}1+X,1+Y,+Z

Table S5 Bond Angles for KNBaN.

Atom	Atom	Atom	Angle/˚	Atom	Atom	Atom	Angle/˚
O1[1]	Nb1	O1	180.00(2)	K1[1]	O2	K1	89.59(3)
O1[1]	Nb1	O2[2]	93(2)	K1[1]	O2	K1[3]	180(4)
O1[1]	Nb1	O2	93(2)	K1[3]	O2	K1	90(4)
O1[1]	Nb1	O3[3]	92(2)	K1[1]	O2	K1[5]	91(4)
O1[1]	Nb1	O3	92(2)	K1[5]	O2	K1[3]	89.59(3)
O1	Nb1	K1[4]	123.7(10)	Na1[5]	O2	Na1[3]	89.59(3)
O1[1]	Nb1	K1[4]	56.3(10)	Na1[1]	O2	Na1[5]	91(4)
O1	Nb1	K1[5]	123.7(10)	Na1[1]	O2	Na1[3]	180(4)
O1[1]	Nb1	K1[5]	56.3(10)	Nb1	O3	Nb1[10]	176(4)
O1[1]	Nb1	Na1[4]	56.3(10)	Nb1[10]	O3	K1	91.3(15)
O1	Nb1	Na1[5]	123.7(10)	Nb1	O3	K1[2]	91.3(15)
O1	Nb1	Na1[4]	123.7(10)	Nb1	O3	K1[4]	88.6(16)
O1	Nb1	Na1[6]	123.7(10)	Nb1	O3	K1[1]	88.7(16)
O1[1]	Nb1	Na1[1]	56.3(10)	Nb1[10]	O3	K1[4]	88.7(16)
O1	Nb1	Na1[1]	123.7(10)	Nb1[10]	O3	K1[2]	91.4(15)
O1[1]	Nb1	Na1[5]	56.3(10)	Nb1[10]	O3	K1[1]	88.6(16)
O1[1]	Nb1	Na1[6]	56.3(10)	Nb1	O3	K1	91.4(15)
O2	Nb1	O1	87(2)	Nb1	O3	Na1[2]	91.3(15)
O2[2]	Nb1	O1	87(2)	Nb1	O3	Na1[1]	88.7(16)
O2	Nb1	O2[2]	175(5)	Nb1[10]	O3	Na1[2]	91.4(15)
O2	Nb1	O3	89.84(14)	Nb1	O3	Na1[4]	88.6(16)
O2[2]	Nb1	O3[3]	89.84(15)	Nb1[10]	O3	Na1[4]	88.7(16)
O2	Nb1	O3[3]	89.98(14)	Nb1[10]	O3	Na1[1]	88.6(16)
O2[2]	Nb1	O3	89.98(15)	K1[4]	O3	K1[2]	89.81(6)
O2[2]	Nb1	K1[5]	127.6(13)	K1[4]	O3	K1[1]	91(4)
O2	Nb1	K1[4]	127.6(13)	K1[1]	O3	K1[2]	179(4)
O2	Nb1	K1[5]	56(2)	K1[2]	O3	K1	89(4)
O2[2]	Nb1	K1[4]	56(2)	K1[1]	O3	K1	89.81(6)
O2[2]	Nb1	Na1[1]	127.7(13)	K1[4]	O3	K1	179(4)
O2	Nb1	Na1[1]	56(2)	Na1[1]	O3	Na1[2]	179(4)
O2[2]	Nb1	Na1[5]	127.6(13)	Na1[4]	O3	Na1[2]	89.81(6)
O2	Nb1	Na1[6]	127.7(13)	Na1[4]	O3	Na1[1]	91(4)
O2[2]	Nb1	Na1[4]	56(2)	O1[10]	K1	O1	90.22(5)
O2	Nb1	Na1[5]	56(2)	O1[10]	K1	O1[11]	89.77(5)
O2	Nb1	Na1[4]	127.6(13)	O1[9]	K1	O1[10]	179(5)
O2[2]	Nb1	Na1[6]	56(2)	O1[9]	K1	O1	89.77(5)
O3[3]	Nb1	O1	88(2)	O1	K1	O1[11]	179(5)
O3	Nb1	O1	88(2)	O1[9]	K1	O1[11]	90.22(6)
O3	Nb1	O3[3]	176(4)	O1[9]	K1	O2[10]	120(3)
O3[3]	Nb1	K1[5]	55.3(18)	O1	K1	O2[10]	120(3)
O3[3]	Nb1	K1[4]	127.4(13)	O1[9]	K1	O2	59.5(14)
O3	Nb1	K1[5]	127.4(13)	O1[10]	K1	O2	120(3)
O3	Nb1	K1[4]	55.3(18)	O1[11]	K1	O2[10]	59.5(14)
O3[3]	Nb1	Na1[1]	127.5(13)	O1[10]	K1	O2[10]	59.5(14)
O3[3]	Nb1	Na1[6]	55.2(18)	O1	K1	O2	59.5(14)
O3[3]	Nb1	Na1[5]	55.3(18)	O1[11]	K1	O2	120(3)
O3[3]	Nb1	Na1[4]	127.4(13)	O1[11]	K1	O3	119(3)

O3	Nb1	Na1[4]	55.3(18)	O1[10]	K1	O3	59.9(16)
O3	Nb1	Na1[5]	127.4(13)	O1[9]	K1	O3[9]	59.9(16)
O3	Nb1	Na1[1]	55.2(18)	O1[9]	K1	O3	119(3)
O3	Nb1	Na1[6]	127.5(13)	O1[11]	K1	O3[9]	59.8(16)
K1[4]	Nb1	K1[5]	112.5(19)	O1	K1	O3	59.8(16)
Na1[6]	Nb1	Na1[1]	112.6(19)	O1[10]	K1	O3[9]	119(3)
Na1[4]	Nb1	Na1[1]	71.9(9)	O1	K1	O3[9]	119(3)
Na1[5]	Nb1	Na1[1]	72.2(9)	O2[7]	K1	O1[11]	121(3)
Na1[5]	Nb1	Na1[6]	71.9(9)	O2[7]	K1	O1	60.1(14)
Na1[4]	Nb1	Na1[5]	112.5(19)	O2[12]	K1	O1[10]	60.2(14)
Na1[4]	Nb1	Na1[6]	72.2(10)	O2[7]	K1	O1[9]	60.2(14)
Nb1[7]	O1	Nb1	180.0	O2[12]	K1	O1	121(3)
Nb1	O1	K1[3]	91(2)	O2[12]	K1	O1[9]	121(3)
Nb1	O1	K1	91(2)	O2[12]	K1	O1[11]	60.1(14)
Nb1	O1	K1[8]	91(2)	O2[7]	K1	O1[10]	121(3)
Nb1[7]	O1	K1	89(2)	O2[7]	K1	O2[10]	180(4)
Nb1[7]	O1	K1[3]	89(2)	O2[10]	K1	O2	90(4)
Nb1[7]	O1	K1[8]	89(2)	O2[7]	K1	O2	89.59(3)
Nb1[7]	O1	K1[2]	89(2)	O2[7]	K1	O2[12]	91(4)
Nb1	O1	K1[2]	91(2)	O2[12]	K1	O2[10]	89.59(3)
Nb1[7]	O1	Na1[2]	89(2)	O2[12]	K1	O2	180(4)
Nb1[7]	O1	Na1[3]	89(2)	O2	K1	O3	60(2)
Nb1	O1	Na1[2]	91(2)	O2[10]	K1	O3	60(2)
Nb1	O1	Na1[8]	91(2)	O2[12]	K1	O3[9]	120.0(9)
Nb1	O1	Na1[3]	91(2)	O2[12]	K1	O3	120.0(9)
Nb1[7]	O1	Na1[8]	89(2)	O2	K1	O3[9]	60(2)
K1[3]	O1	K1[2]	179(5)	O2[7]	K1	O3	120.0(9)
K1[8]	O1	K1	179(5)	O2[10]	K1	O3[9]	60(2)
K1[2]	O1	K1	89.77(5)	O2[7]	K1	O3[9]	120.0(9)
K1[3]	O1	K1[8]	89.77(5)	O3[7]	K1	O1	60.1(16)
K1[2]	O1	K1[8]	90.22(6)	O3[13]	K1	O1[10]	121(3)
K1[3]	O1	K1	90.22(7)	O3[13]	K1	O1	121(3)
Na1[3]	O1	Na1[8]	89.77(5)	O3[13]	K1	O1[9]	60.2(16)
Na1[2]	O1	Na1[8]	90.22(6)	O3[7]	K1	O1[11]	121(3)
Na1[3]	O1	Na1[2]	179(5)	O3[7]	K1	O1[10]	60.2(16)
Nb1	O2	Nb1[9]	175(5)	O3[13]	K1	O1[11]	60.1(16)
Nb1[9]	O2	K1[1]	88.2(17)	O3[7]	K1	O1[9]	121(3)
Nb1	O2	K1[1]	88.3(17)	O3[7]	K1	O2[12]	61(2)
Nb1[9]	O2	K1[3]	91.9(16)	O3[7]	K1	O2[10]	119.7(9)
Nb1	O2	K1[5]	88.2(17)	O3[7]	K1	O2[7]	61(2)
Nb1[9]	O2	K1	91.8(16)	O3[13]	K1	O2[10]	119.6(9)
Nb1[9]	O2	K1[5]	88.3(17)	O3[13]	K1	O2[12]	61(2)
Nb1	O2	K1	91.9(16)	O3[7]	K1	O2	119.6(9)
Nb1	O2	K1[3]	91.8(16)	O3[13]	K1	O2	119.7(9)
Nb1[9]	O2	Na1[1]	88.2(17)	O3[13]	K1	O2[7]	61(2)
Nb1[9]	O2	Na1[3]	91.9(16)	O3[7]	K1	O3[9]	179(4)
Nb1	O2	Na1[3]	91.8(16)	O3[13]	K1	O3[9]	89.81(6)
Nb1	O2	Na1[1]	88.3(17)	O3[13]	K1	O3	179(4)
Nb1[9]	O2	Na1[5]	88.3(17)	O3[13]	K1	O3[7]	91(4)
Nb1	O2	Na1[5]	88.2(17)	O3[7]	K1	O3	89.81(6)

K1[5]	O2	K1	180(4)	O3[9]	K1	O3	89(4)

[1]+X,-1+Y,+Z; [2]-1+X,+Y,+Z; [3]+X,+Y,1+Z; [4]-1+X,-1+Y,+Z; [5]+X,-1+Y,1+Z; [6]-1+X,-1+Y,1+Z; [7]+X,1+Y,+Z; [8]-1+X,+Y,1+Z; [9]1+X,+Y,+Z; [10]+X,+Y,-1+Z; [11]1+X,+Y,-1+Z; [12]+X,1+Y,-1+Z; [13]1+X,1+Y,+Z

Table S6 Atomic Occupancy for KNBaN.

Atom	Occupancy	Atom	Occupancy
K1	0.5	Na1	0.5

Table S7 Crystal data and structure refinement for a 98.5 mol % ($K_{0.5}Na_{0.5}$)NbO_3-1.5 mol % Ba($Cu_{0.13}Nb_{0.66}$)O_3 single crystal grown on a [001] $KTaO_3$ seed crystal by sintering at 1125°C for 10 h (KNBaCuN).

Identification code	KNBaCuN
**Empirical formula**	$K_{0.5}Na_{0.5}NbO_3$
**Empirical formula**	$K_{0.5}Na_{0.5}NbO_3$
**Formula weight**	171.96
**Temperature/K**	296.15
**Crystal system**	monoclinic
**Space group**	P2
**a/Å**	3.9714(6)
**b/Å**	3.9683(5)
**c/Å**	3.9996(6)
**α/°**	90
**β/°**	89.999(9)
**γ/°**	90
**Volume/Å^3**	63.033(16)
**Z**	1
**ρ_{calc}g/cm^3**	4.530
**μ/mm^{-1}**	5.411
**F(000)**	80.0
**Crystal size/mm^3**	0.15 × 0.15 × 0.1
**Radiation**	MoKα (λ = 0.71073)
**2Θ range for data collection/°**	10.194 to 49.582
**Index ranges**	$-4 \le h \le 4, -4 \le k \le 4, -4 \le l \le 4$
**Reflections collected**	705
**Independent reflections**	234 [R_{int} = 0.0247, R_{sigma} = 0.0227]
**Data/restraints/parameters**	234/31/26
**Goodness-of-fit on F^2**	1.028
**Final R indexes [I>=2σ (I)]**	R_1 = 0.0248, wR_2 = 0.0685
**Final R indexes [all data]**	R_1 = 0.0248, wR_2 = 0.0685
**Largest diff. peak/hole / e Å^{-3}**	0.67/-1.38

Table S8 Fractional Atomic Coordinates (×10^4) and Equivalent Isotropic Displacement Parameters (Å^2×10^3) for KNBaCuN. U_{eq} is defined as 1/3 of the trace of the orthogonalised U_{IJ} tensor.

Atom	x	y	z	U(eq)
Nb1	0	8821.9(2)	5000	4.1(5)
O1	5000	9050(150)	5000	30(5)
O2	0	14030(140)	5000	27(5)
O3	0	9050(150)	0	29(5)
K1	5000	14060(110)	0	11(3)
Na1	5000	14060(110)	0	11(3)

Table S9 Anisotropic Displacement Parameters (Å^2×10^3) for KNBaCuN. The Anisotropic displacement factor exponent takes the form: $-2\pi^2[h^2a^{*2}U_{11}+2hka^*b^*U_{12}+\ldots]$.

Atom	U_{11}	U_{22}	U_{33}	U_{23}	U_{13}	U_{12}
Nb1	4.0(6)	4.7(11)	3.5(6)	0	0.5(4)	0
O1	31(6)	14(13)	45(6)	0	0(5)	0
O2	32(6)	8(12)	43(6)	0	-2(5)	0
O3	29(5)	13(13)	44(6)	0	1(5)	0
K1	15.2(19)	2(10)	16.3(19)	0	-0.3(14)	0
Na1	15.2(19)	2(10)	16.3(19)	0	-0.3(14)	0

Table S10 Bond Lengths for KNBaCuN.

Atom	Atom	Length/Å	Atom	Atom	Length/Å
Nb1	O1	1.988(3)	O2	K1[9]	2.8182(6)
Nb1	O1[1]	1.988(3)	O2	K1[1]	2.8182(6)
Nb1	O2[2]	1.90(6)	O2	Na1[1]	2.8182(6)
Nb1	O2	2.07(6)	O2	Na1[9]	2.8182(6)
Nb1	O3[3]	2.002(3)	O2	Na1[3]	2.8182(6)
Nb1	O3	2.002(3)	O3	Nb1[10]	2.002(3)
Nb1	K1[4]	3.39(2)	O3	K1	2.81(6)
Nb1	K1[2]	3.39(2)	O3	K1[1]	2.81(6)
Nb1	K1[5]	3.39(2)	O3	K1[5]	2.80(6)
Nb1	Na1[2]	3.39(2)	O3	K1[2]	2.80(6)
Nb1	Na1[4]	3.39(2)	O3	Na1[1]	2.81(6)
Nb1	Na1[5]	3.39(2)	O3	Na1[2]	2.80(6)
O1	Nb1[6]	1.988(3)	O3	Na1[5]	2.80(6)
O1	K1	2.82(6)	K1	O1[10]	2.82(6)
O1	K1[7]	2.81(6)	K1	O1[11]	2.81(6)
O1	K1[3]	2.82(6)	K1	O1[8]	2.81(6)
O1	K1[2]	2.81(6)	K1	O2[6]	2.8182(6)
O1	Na1[2]	2.81(6)	K1	O2[10]	2.8182(6)
O1	Na1[7]	2.81(6)	K1	O2[12]	2.8182(6)
O1	Na1[3]	2.82(6)	K1	O3[8]	2.80(6)
O2	Nb1[8]	1.90(6)	K1	O3[6]	2.81(6)
O2	K1[3]	2.8182(6)	K1	O3[13]	2.80(6)
O2	K1	2.8182(6)			

[1]-1+X,+Y,+Z; [2]+X,-1+Y,+Z; [3]+X,+Y,1+Z; [4]-1+X,-1+Y,1+Z; [5]-1+X,-1+Y,+Z; [6]1+X,+Y,+Z; [7]+X,-1+Y,1+Z; [8]+X,1+Y,+Z; [9]-1+X,+Y,1+Z; [10]+X,+Y,-1+Z; [11]+X,1+Y,-1+Z; [12]1+X,+Y,-1+Z; [13]1+X,1+Y,+Z

Table S11 Bond Angles for KNBaCuN.

Atom	Atom	Atom	Angle/˚	Atom	Atom	Atom	Angle/˚
O1	Nb1	O1[1]	175(3)	K1	O2	K1[1]	89.594(19)
O1[1]	Nb1	O2	87.4(17)	K1[9]	O2	K1[1]	90.40(3)
O1	Nb1	O2	87.4(17)	K1[9]	O2	K1[2]	89.59(2)
O1	Nb1	O3[2]	89.88(12)	K1[2]	O2	K1[1]	180(3)
O1	Nb1	O3	89.89(12)	Na1[9]	O2	Na1[2]	89.59(2)
O1[1]	Nb1	O3	89.88(11)	Na1[9]	O2	Na1[1]	90.40(3)
O1[1]	Nb1	O3[2]	89.89(12)	Na1[2]	O2	Na1[1]	180(3)
O1[1]	Nb1	K1[3]	127.6(10)	Nb1	O3	Nb1[10]	175(3)
O1	Nb1	K1[4]	127.6(10)	Nb1	O3	K1	91.8(12)
O1[1]	Nb1	K1[4]	56.0(14)	Nb1	O3	K1[4]	88.2(12)
O1	Nb1	K1[3]	56.0(14)	Nb1[10]	O3	K1	91.8(12)
O1	Nb1	K1[5]	127.6(10)	Nb1[10]	O3	K1[3]	88.2(12)
O1[1]	Nb1	K1[5]	56.0(14)	Nb1	O3	K1[3]	88.2(12)
O1[1]	Nb1	Na1[3]	127.6(10)	Nb1[10]	O3	K1[4]	88.2(12)
O1	Nb1	Na1[3]	56.0(14)	Nb1[10]	O3	K1[1]	91.8(12)
O1[1]	Nb1	Na1[5]	56.0(14)	Nb1	O3	K1[1]	91.8(12)
O1	Nb1	Na1[5]	127.6(10)	Nb1	O3	Na1[1]	91.8(12)
O1[1]	Nb1	Na1[4]	56.0(14)	Nb1[10]	O3	Na1[3]	88.2(12)
O1	Nb1	Na1[4]	127.6(10)	Nb1[10]	O3	Na1[1]	91.8(12)
O2[3]	Nb1	O1	92.6(17)	Nb1[10]	O3	Na1[4]	88.2(12)
O2[3]	Nb1	O1[1]	92.6(17)	Nb1	O3	Na1[4]	88.2(12)
O2[3]	Nb1	O2	180.00(2)	Nb1	O3	Na1[3]	88.2(12)
O2[3]	Nb1	O3[2]	92.5(17)	K1[3]	O3	K1[1]	180(3)
O2[3]	Nb1	O3	92.5(17)	K1[4]	O3	K1[3]	90(3)
O2[3]	Nb1	K1[3]	56.2(6)	K1[1]	O3	K1	90(3)
O2	Nb1	K1[3]	123.8(6)	K1[3]	O3	K1	89.955(12)
O2[3]	Nb1	K1[5]	56.2(6)	K1[4]	O3	K1	180(3)
O2[3]	Nb1	K1[4]	56.2(6)	K1[4]	O3	K1[1]	89.955(13)
O2	Nb1	K1[4]	123.8(6)	Na1[4]	O3	Na1[1]	89.955(13)
O2	Nb1	K1[5]	123.8(6)	Na1[4]	O3	Na1[3]	90(3)
O2[3]	Nb1	Na1[3]	56.2(6)	Na1[3]	O3	Na1[1]	180(3)
O2	Nb1	Na1[3]	123.8(6)	O1[8]	K1	O1[10]	180(3)
O2[3]	Nb1	Na1[5]	56.2(6)	O1[10]	K1	O1	90(3)
O2	Nb1	Na1[5]	123.8(6)	O1[11]	K1	O1	180(3)
O2[3]	Nb1	Na1[4]	56.2(6)	O1[11]	K1	O1[10]	89.550(14)
O2	Nb1	Na1[4]	123.8(6)	O1[8]	K1	O1[11]	91(3)
O3	Nb1	O2	87.5(17)	O1[8]	K1	O1	89.550(16)
O3[2]	Nb1	O2	87.5(17)	O1[11]	K1	O2	120.4(19)
O3	Nb1	O3[2]	175(3)	O1[8]	K1	O2[6]	59.9(12)
O3	Nb1	K1[3]	55.7(14)	O1[8]	K1	O2[10]	120.4(19)
O3[2]	Nb1	K1[3]	127.8(10)	O1[8]	K1	O2	59.9(12)
O3	Nb1	K1[4]	55.7(14)	O1[11]	K1	O2[6]	120.4(18)
O3[2]	Nb1	K1[4]	127.8(10)	O1[8]	K1	O2[12]	120.4(19)

O3[2]	Nb1	K1[5]	55.7(14)	O1[11]	K1	O2[12]	59.9(12)
O3	Nb1	K1[5]	127.8(10)	O1[11]	K1	O2[10]	59.9(12)
O3[2]	Nb1	Na1[4]	127.8(10)	O2[10]	K1	O1	120.0(19)
O3[2]	Nb1	Na1[3]	127.8(10)	O2[6]	K1	O1	59.6(12)
O3	Nb1	Na1[5]	127.8(10)	O2[12]	K1	O1	120.0(19)
O3	Nb1	Na1[3]	55.7(14)	O2[10]	K1	O1[10]	59.6(12)
O3[2]	Nb1	Na1[5]	55.7(14)	O2[6]	K1	O1[10]	120.0(19)
O3	Nb1	Na1[4]	55.7(14)	O2	K1	O1	59.6(12)
K1[5]	Nb1	K1[4]	72.2(6)	O2	K1	O1[10]	120.0(19)
K1[5]	Nb1	K1[3]	112.3(12)	O2[12]	K1	O1[10]	59.6(12)
K1[3]	Nb1	K1[4]	71.6(6)	O2	K1	O2[6]	89.59(2)
Na1[3]	Nb1	Na1[4]	71.6(6)	O2	K1	O2[10]	90.40(3)
Na1[5]	Nb1	Na1[4]	72.2(6)	O2	K1	O2[12]	180(3)
Na1[5]	Nb1	Na1[3]	112.3(12)	O2[6]	K1	O2[10]	180(3)
Nb1	O1	Nb1[6]	175(3)	O2[12]	K1	O2[10]	89.59(2)
Nb1[6]	O1	K1[7]	88.2(12)	O2[12]	K1	O2[6]	90.40(2)
Nb1[6]	O1	K1[3]	88.2(12)	O3	K1	O1	60.1(13)
Nb1[6]	O1	K1[2]	91.8(12)	O3[13]	K1	O1[8]	60.2(13)
Nb1	O1	K1	91.8(12)	O3[6]	K1	O1	60.1(13)
Nb1	O1	K1[7]	88.2(12)	O3[8]	K1	O1[11]	60.2(13)
Nb1	O1	K1[3]	88.2(12)	O3	K1	O1[8]	119.9(7)
Nb1	O1	K1[2]	91.8(12)	O3[13]	K1	O1	119.8(7)
Nb1[6]	O1	K1	91.8(12)	O3[13]	K1	O1[10]	119.8(7)
Nb1	O1	Na1[2]	91.8(12)	O3[8]	K1	O1[8]	60.2(13)
Nb1	O1	Na1[7]	88.2(12)	O3[8]	K1	O1[10]	119.8(7)
Nb1[6]	O1	Na1[2]	91.8(12)	O3[13]	K1	O1[11]	60.2(13)
Nb1	O1	Na1[3]	88.2(12)	O3[6]	K1	O1[10]	60.1(13)
Nb1[6]	O1	Na1[7]	88.2(12)	O3[8]	K1	O1	119.8(7)
Nb1[6]	O1	Na1[3]	88.2(12)	O3	K1	O1[11]	119.9(7)
K1[2]	O1	K1	90(3)	O3[6]	K1	O1[8]	119.9(7)
K1[7]	O1	K1[2]	89.550(14)	O3[6]	K1	O1[11]	119.9(7)
K1[3]	O1	K1[7]	91(3)	O3	K1	O1[10]	60.1(13)
K1[3]	O1	K1[2]	180(3)	O3[6]	K1	O2[12]	60.0(12)
K1[3]	O1	K1	89.550(12)	O3[13]	K1	O2[6]	60.2(12)
K1[7]	O1	K1	180(3)	O3[6]	K1	O2[10]	119.7(19)
Na1[7]	O1	Na1[2]	89.550(14)	O3[6]	K1	O2	119.7(19)
Na1[3]	O1	Na1[2]	180(3)	O3[8]	K1	O2[10]	60.2(12)
Na1[3]	O1	Na1[7]	91(3)	O3	K1	O2[6]	119.7(19)
Nb1[8]	O2	Nb1	180.0	O3[8]	K1	O2	60.2(12)
Nb1[8]	O2	K1[1]	89.8(17)	O3	K1	O2[12]	119.7(19)
Nb1[8]	O2	K1	89.8(17)	O3[13]	K1	O2[12]	60.2(12)
Nb1[8]	O2	K1[2]	89.8(17)	O3[8]	K1	O2[6]	120.1(18)
Nb1	O2	K1	90.2(17)	O3	K1	O2	60.0(12)
Nb1	O2	K1[2]	90.2(17)	O3[13]	K1	O2	120.1(19)
Nb1	O2	K1[9]	90.2(17)	O3[8]	K1	O2[12]	120.1(18)
Nb1	O2	K1[1]	90.2(17)	O3	K1	O2[10]	60.0(12)
Nb1[8]	O2	K1[9]	89.8(17)	O3[13]	K1	O2[10]	120.1(19)
Nb1[8]	O2	Na1[9]	89.8(17)	O3[6]	K1	O2[6]	60.0(12)
Nb1[8]	O2	Na1[1]	89.8(17)	O3[13]	K1	O3[8]	90(3)
Nb1	O2	Na1[9]	90.2(17)	O3[6]	K1	O3	90(3)

Nb1	O2	$Na1^{2}$	90.2(17)	$O3^{13}$	K1	$O3^{6}$	89.955(14)
$Nb1^{8}$	O2	$Na1^{2}$	89.8(17)	$O3^{8}$	K1	O3	89.955(14)
Nb1	O2	$Na1^{1}$	90.2(17)	$O3^{8}$	K1	$O3^{6}$	180(3)
K1	O2	$K1^{9}$	180(3)	$O3^{13}$	K1	O3	180(3)
K1	O2	$K1^{2}$	90.40(3)				

^{1}-1+X,+Y,+Z; ^{2}+X,+Y,1+Z; ^{3}+X,-1+Y,+Z; ^{4}-1+X,-1+Y,+Z; ^{5}-1+X,-1+Y,1+Z; ^{6}1+X,+Y,+Z; ^{7}+X,-1+Y,1+Z; ^{8}+X,1+Y,+Z; ^{9}-1+X,+Y,1+Z; ^{10}+X,+Y,-1+Z; ^{11}+X,1+Y,-1+Z; ^{12}1+X,+Y,-1+Z; ^{13}1+X,1+Y,+Z

Table S12 Atomic Occupancy for KNBaCuN.

Atom	Occupancy	Atom	Occupancy
K1	0.5	Na1	0.5
